# Diabetic macular edema (DME): dissecting pathogenesis, prognostication, diagnostic modalities along with current and futuristic therapeutic insights

**DOI:** 10.1186/s40942-024-00603-y

**Published:** 2024-10-28

**Authors:** Ahmed Sermed Al Sakini, Abdulrahman Khaldoon Hamid, Zainab A. Alkhuzaie, Sandra Thair Al-Aish, Shahad Al-Zubaidi, Abduljaber A’Ed Tayem, Mohammed Ayad Alobi, Anne Sermed Al Sakini, Rami Thair Al-Aish, Khayry Al-Shami, Hamdah Hanifa, Sara S. Khunda

**Affiliations:** 1 Department of Surgery, College of Medicine, University of Baghdad, Baghdad, Iraq; 2 College of Medicine, University of Alexandria, Alexandria, Egypt; 3 Clinical Teaching Fellow, College of Medicine, University of Kufa, Al-Najaf, Iraq; 4 Clinical Teaching Fellow, University of Baghdad, Al-Kindy Medical College, Baghdad, Iraq; 5 College of Medicine, University of Jordan, Amman, Jordan; 6 Department of Internal Medicine , Ibn Sina Teaching Hospital, Mosul, Iraq; 7 Department of Clinical Medical Sciences, Faculty of Medicine, Yarmouk University, Irbid, Jordan; 8 Faculty of Medicine, University of Kalamoon, Al-Nabk, Syria; 9 Department of Internal Medicine, Baghdad Medical City, Baghdad, Iraq

**Keywords:** Diabetic macular edema, Anti-VEGFs, Diabetes mellitus, Proliferative diabetic retinopathy, Selective retinal therapy

## Abstract

One of the most common health concerns disturbing people within working years globally is diabetes mellitus (DM). One well-known consequence of DM is vascular damage, which can manifest as macro- and microangiopathy affecting the ocular retina. Therefore, Diabetic macular edema (DME) is a major sight-threatening complication of diabetic retinopathy (DR) worldwide. It is the most prevalent cause of significant vision impairment in diabetic patients. Long-term vision loss can be avoided by following early DME treatment guidelines in everyday life. Hence, there are various therapeutic approaches for DME management. Currently, the first-line treatment for DME is anti-VEGF family drugs, such as ranibizumab, brolucizumab, bevacizumab, and aflibercept. Nevertheless, relapses of the disease, inadequate response, and resistance during anti-VEGF therapy are still seen because of the intricate pathophysiological foundation of the disease. Consequently, there is an excellent requirement for therapeutic approaches to advance and become better at controlling diseases more satisfactorily and require fewer treatments overall. We conducted a thorough literature search in the current review to present a comprehensive overview of the primary data about the current DME therapeutic agents. We also covered the novel advances in DME management and probable future treatments being investigated and developed. This review recommended that Large clinical trials should afford sufficient evidence to support these innovative treatment modalities.

## Background

Diabetic macular edema (DME) is one of the significant causes of irreversible visual impairment and subsequent permanent vision loss and blindness. It is currently on an exponential rise due to its prevalence in patients with diabetes mellitus type 2, which is considered a global epidemic of the modern era with about 382 million individuals currently affected worldwide, expected to reach 592 million by the year 2035 and 783.2 million in 2045, this increase is mainly attributed to the rising prevalence of obesity and prolongation of the average human life expectancy [1, 2]. It was found that about 10.2% of 22,896 diabetic patients had diabetic retinopathy, and 6.81% had DME [3]. DME is a term used to describe retinal thickening in or around the center of the macula. This persistent macular edema primarily affects and damages the retinal neuromuscular system, leading to diabetic retinopathy and disturbed visual acuity. This edema is mainly mediated by the expression of vascular endothelial growth factor (VEGF), which leads to increased permeability, obstruction and damage of the retinal capillaries, resulting in serous blood components leakages and hemorrhages and, ultimately, failure of the neurovascular units (NVUs) of the retina [4, 5]. 

Early detection of these changes by periodic checkups of diabetic patients with optical coherence tomography (OCT) is crucial in setting up effective measures for DME prevention, early management and control. These measures include hyperglycemia and blood pressure control and Specific ophthalmic treatments such as intravitreal anti-VEGF drug injections, intravitreal corticosteroid injections, focal laser photocoagulation, and vitrectomy [2, 4, 6]. Although the introduction of anti-VEGF has revolutionized our understanding of DME, there are still so many cases resistant to treatment, with many responding to steroids, all further support the idea of DME being of multifactorial origin [6, 7]. In this narrative, the authors thoroughly review the epidemiology, pathophysiology, diagnostic modalities, and current and promising frontiers for treating patients with DME.

### Pathophysiology of DME and DR

Patients usually remain asymptomatic for years before the typical manifestations of DR start to take place. They can be clinically diagnosed, making early diagnosis of the disease complex for apparently healthy individuals. However, it has been objectively accepted that many noticeable changes occur during the disease’s asymptomatic phase in the retina, especially at the periphery, affecting the retinal, endothelial, and ganglionic cells in the eye, resulting in gradual cellular and functional disability. These changes are also a critical reflective indicator of systemic diabetic microvascular complications [8]. 

As a result of the insufficient action of insulin in a diabetic patient, Glucose tends to build up in the bloodstream, leading to hyperglycemia. It alters the membrane permeability and elasticity of blood vessels, resulting in micro-aneurysms, microscopic hemorrhages, deposition of hard exudates and endothelial and pericyte cell damage resulting from a chronically high glycemic index, mainly in uncontrolled diabetic patients. Still, it also occurs with controlled DM as a long-term, slowly developing complication [9]. 

Proliferative Diabetic Retinopathy is the advanced stage of the disease; it is characterized by neovascularization and increased severity of symptoms from NPDR.

As the micro-vascular pathology and damage in the capillary beds of the retina progresses, there will be resultant hypoxia of the retina due to lack of blood supply, hyperglycemia along with hypoxia lead to the release of cytokines, chemokines which result in inflammation and glial cell activation, and release of vascular growth factors most notably VEGF, to restore circulation by forming new vessels on the optic disc and elsewhere on the retina, in a process termed neovascularization [9]. 

VEGF and the other released growth factors and proinflammatory molecules induce a decrease in the vascular adhesion molecules VCAMs between the endothelial cells, an increase in subclinical inflammation and production of nitric oxide resulting in vasodilation, and the upregulation of multiple metabolic pathways that ultimately result in increased endoplasmic reticulum stress and the accumulation of Reactive Oxygen Species (ROS) thus disrupting the cellular activities and damaging the DNA [10]. 

Therefore, markers like VCAMs, VEGF and erythropoietin, among others, show increased levels in the blood with the progression of clinical and subclinical retinopathy; these markers provide promising targets for early diagnosis, treatment, and monitoring of disease severity [11]. 

As a result, it can lead to increased retinal thickness, maculopathy and macular edema, neurodegeneration and further damage to the blood-retinal barrier [12]. 

Diabetic maculopathy can be defined as the presence of DR in and around the macula, resulting in diabetic macular edema that is focal or diffuse, accumulation of hard exudates, micro-hemorrhages and retinal thickening in the macula. DME can start developing at any stage of the disease but is usually associated with more advanced presentations, as in severe NPDR and PDR, it is considered the leading cause of vision loss among DR pathologies [13].

All these factors contribute to the symptoms observed in patients of PDR, as they usually present with progressively worsening vision or sudden blindness, scotomas and other shapes in the field of vision, redness, sore eyes and even lead to the advanced presentations of PDR like vitreous hemorrhages and retinal detachments involving or sparing the macula [14]. 

Risk factors for DR can be either physiological, as in pregnancy, obesity or puberty. Or pathological, as in High BP, Kidney disease, Uncontrolled T1DM, Oxidative stress and poor levels of lipids, proteins, vitamin D or glucose in the blood. Chronic hyperglycemia is the most important and predictive factor for both the progression and control of the disease, as it was clearly shown in many trials that very intensive glycemic control can reduce the incidence of DR by up to 62–85% [15, 16].

**Fig. 1:**
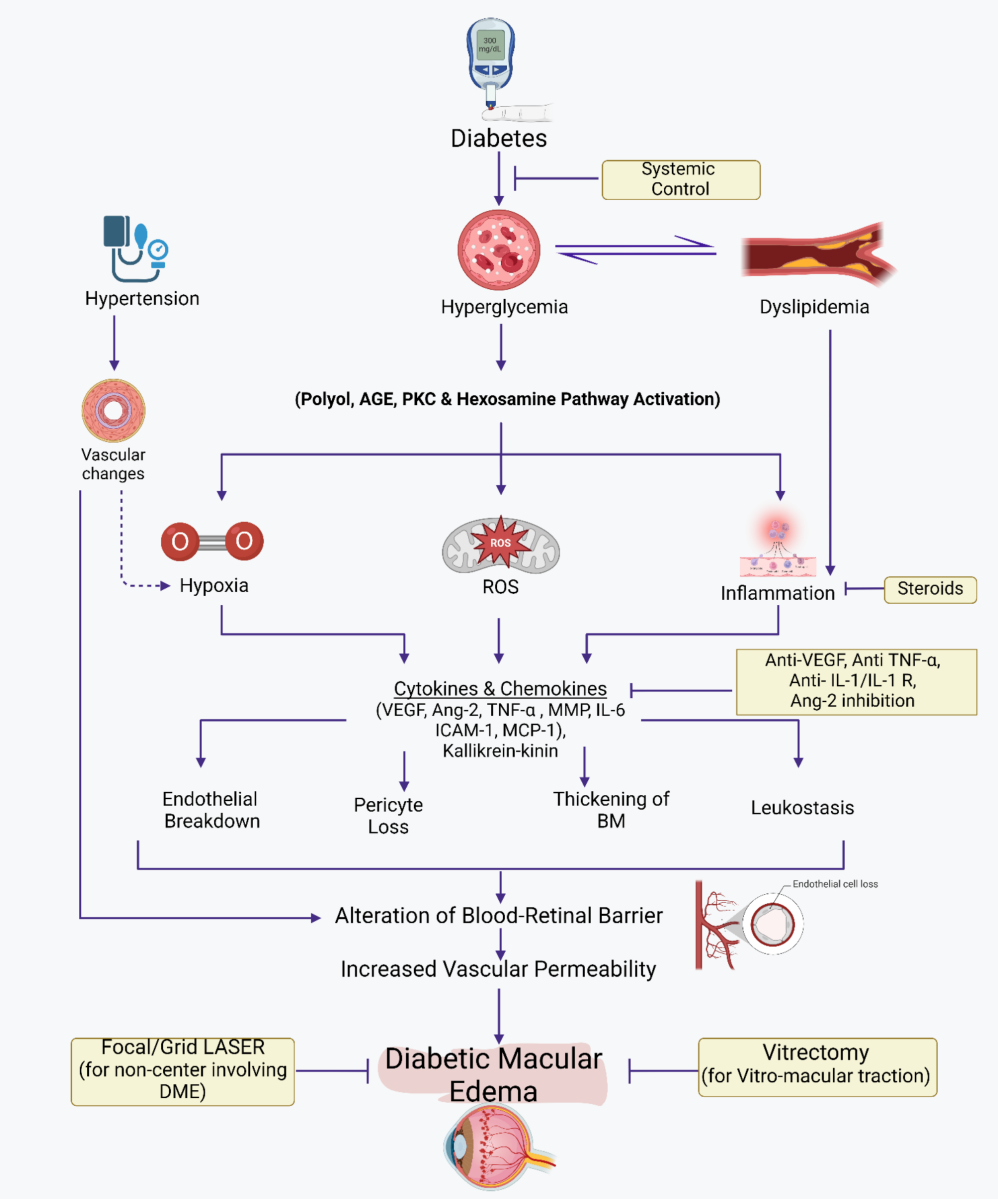
The pathogenesis of diabetic macular edema and management strategies. Illustrates the progression and effects of DME. It begins with conditions such as Hyperglycemia which could be associated with aggravating factors (i.e. hypertension), leading to Biochemical and Molecular Abnormalities. These abnormalities trigger a series of cellular and molecular reactions, including the generation of Reactive Oxygen Species (ROS) and its effects. These effects include inflammation, endothelial cell junction breakdown, pericyte loss, thickening of the Basement Membrane (BM), and leukostasis. These processes alter the blood-retinal barrier and increase vascular permeability, resulting in Diabetic Macular Edema. The figure indicates current available treatments like Anti-VEGFs, Steroids, Systemic treatments, Focal/Grid Laser (for DME involving the center) and Vitrectomy (for Vitreo-macular traction).

More Recent studies have shown that endothelial nitric oxide synthase eNOS gene polymorphisms may play a significant role in the upregulation of eNOS, especially in the Caucasian population, triggering an increased production of nitric oxide, which is an essential factor in the elevated levels of Superoxides responsible for the significant damage to the retinal microvasculature. Therefore, research on eNOS gene polymorphisms on chromosome 7q35-36 and its relation to DR suggests that it could be a crucial marker for the early diagnosis of retinal disease in the Western world [17] (see Fig. 1).

### Prognosis and life quality

Long-term visual acuity may benefit from early DME treatment with rigorous therapy before alterations in the outer retinal layers occur. Still, late-treated DME eyes require more injections and focal laser treatments than early-treated eyes. Real-life adherence to early DME therapy will help avoid long-term visual loss [18]. On the other hand, timely treatment can help preserve residual vision even in individuals who present with visual loss later in life [19]. However, the influence of gender on VTDR was only slightly significant, whereas the effect of age on VTDR and CSME prevalence was not statistically significant [20]. DME can result in permanent. Retinal alterations and chronic edema if treatment is not received, which would compromise vision. According to studies, 20–30% of DME patients will lose at least three lines of vision within three years if the disease is not treated [21]. Patients may refuse treatment for several reasons, such as mild-to-moderate vision loss, a deranged glucose level, fear of repeated injections and procedure-related complications, incapacity to attend on-schedule and regular follow-up appointments, and, most importantly, treatment costs [22]. Controlling systemic risk factors, such as strict management of hyperglycemia, hyperlipidemia, and hypertension, should be viewed as the cornerstone approach for the prevention and treatment of DR and DME, as DME is still a frequent DR consequence brought on by diabetes [23, 24].

### Current diagnostic modalities

Fluorescein angiography is the most widely used ophthalmological screening test for the detection of microvascular abnormalities in the retina and the choroid, where the color fundus mydriatic **7 Standard Field** 7SF 30° has been regarded as the first-line gold standard retinal examination method for detection of DR as defined by the **ETDRS** [25, 26]. During the procedure, seven different fields are taken with the fundus camera through a contact lens, Providing maximal sensitivity and specificity for diagnosis. As a result, this procedure can be time-consuming for the patient and highly dependent on the reader’s skill and compliance of the patient. Therefore, including fewer fields is often more practical and attractive to use in a clinical setting; this comes with the obvious drawback of decreased field of view. They decreased sensitivity and selectivity to 78% and 86%, respectively, for single-field and down to 92% and 97% for 3-field fundus photography, showing relatively high agreement with 7SF in detecting clinical DR. The 7SF and < 7SF provide limited exposure to the peripheral retina. Hence, it has limited use for the early detection of DR compared to other tests [25, 26].

The ultra-wide field is another imaging technology that utilizes confocal laser scanning ophthalmoscopy to produce high-quality images with much broader peripheral retinal coverage, enabling visualization of up to 200° in a single frame. It is now well-documented that peripheral retinal abnormalities are one of the first signs of asymptomatic damage to the retina. Therefore, UWF has become more valuable in the early detection of DR than 7SF. Mydriatic-UWF has also shown high to near-perfect agreement with 7SF in diagnosing PDR and DME. As a result, UWF has recently been considered the primary screening device for diagnosis of DR. However, 7SF is still much more cost-effective, especially in developing nations [27, 28]. 

Along with the continued rise in cases and relevance of DR in the world, Cost-effective diagnostic modalities make for an attractive goal for the systematic detection in the populace, with methods like non-mydriatic Tele-retinal technology being more easily accessible, cost-effective, and decrease the rising overload on the medical staff. Another candidate for diagnosis is Budget smartphone-based fundus cameras for retinal photography; compared to routine evaluation, these methods are associated with a lower overall sensitivity and high specificity for the detection of retinopathy, being more sensitive and selective for more severe disease presentations like PDR and severe NPDR compared to mild and subclinical NPDR. Therefore, it provides valuable prospects for detecting DR, especially in developing countries [29, 30]. 

Optical Coherence Tomography: is a non-invasive cross-sectional diagnostic method that relies on the exposure of infrared radiation on the retina and measuring the reflection delay time, thereby constructing high-quality 2 and 3-dimensional sections of the retina, providing much more sensitive evaluation of DME and retinal thickness, this method mitigates one of the significant drawbacks of fundus photography; the inability to accurately assess the extent and severity of DME and central foveal thickening, resulting in a lack of essential prognostic markers that are crucial in the treatment decision [31]. 

OCT provides an objective optical biopsy of the retina without the need for skilled operators; it offers a comparatively shorter examination time and is less invasive and better tolerated compared to fundus photography; the increased sensitivity and selectivity in DME assessment observed with OCT make it the primary method for early and definitive diagnosis. Moreover, it also compares favorably with UWF in diagnosing DR pathologies [32]. 

### Methods

#### Literature search strategy

We piloted a broad literature search to conduct a comprehensive narrative review on DME until the 15th of February, 2024. The exploration was accomplished through several electronic databases, including PubMed, Scopus, Embase, and Web of Science, to identify relevant publications, including research articles, reviews, clinical trials, and case reports published up to the current date. The authors employed a combination of keywords and Medical Subject Headings (MeSH) terms such as “Diabetic Macular Edema,” “Diabetic Retinopathy,” “DME Management,” “DME diagnosis,” “Anti-VEGFs for DME,” “DME pathogenesis,” “DME treatment,” and correlated terms. A variety of search terms were used to gather a wide range of literature that is pertinent to the objectives of the review. The search focused on the underlying pathophysiology, epidemiological and prognostic trends, available diagnostic tools, currently approved treatments, advantages and limitations, and the up-to-date advancements in treatment options for diabetic macular edema (DME). Titles and abstracts were carefully reviewed to ensure they aligned with the purposes of the review. This was followed by a comprehensive analysis of the full-text articles to determine if they met the criteria for inclusion. Excluded from the review were publications that were duplicates or did not meet the inclusion criteria.

#### Inclusion and exclusion criteria

Inclusion and exclusion criteria were established to guide the selection of literature for review.

The inclusion criteria were:

- Relevance to the topic and content related to diabetic macular edema (DME) pathophysiology, prognostics, diagnosis, and therapeutic updates.
- Only peer-reviewed, English-language articles were included.
- Studies published before the knowledge cutoff date of 15th February 2024 were included.
- Only studies with available full-text access were included.

The exclusion criteria were:

- Articles were not relevant to the purpose of our review.
- Duplicates or unavailable in full-text format.
- Articles and reports not written in English were excluded.

## Results of current DME treatments

### Anti-VEGFs

#### Ranibizumab

Ranibizumab, also known as Lucentis, is the first anti-VEGF to receive FDA approval for the treatment of Diabetic macular edema(DME) in August 2012, based on results of phase III RISE and RIDE clinical trials which showed 44.8% of patients with DME gained ≥ 15 letters in comparison with 18% in control patients receiving sham injections [33]. In addition to proven efficacy in Protocol I [34, 35], RESTORE phase III trial [36], and other real world and RCTs [37, 38]. Ranibizumab is the Fab fragment of humanized monoclonal IgG, and it acts by blocking VEGF-A. Thereby preventing the breakdown of the blood-retinal barrier, leakage of blood vessels and neovascularization. It is also used in the treatment of wet AMD (wAMD), macular edema caused by retinal vein occlusion (RVO), diabetic retinopathy, Myopic Choroidal Neovascularization (CNV) and retinal ischemia [33, 39]. 

Ranibizumab intravitreal injections (IVR) come in 0.3 mg and 0.5 mg vial dosages given under local anesthesia or via Prefilled single-use syringes, which have been recently introduced. The recommended dose for treating DME is 0.3 mg intravitreal injections each month [33]. Given the need for repeated injections of ranibizumab in various treatment regimens to meet and maintain desirable outcomes, administration of ranibizumab through portal delivery system (PDS) was approved to be used instead for treatment of wet AMD to ensure continuous delivery of ranibizumab with possible same effect of traditional ranibizumab injections [40, 41] adding additional benefit of minimizing subjection to multiple injections thus reducing treatment burden and improve patients satisfaction.

Several factors have been found to influence the variability of response to the treatment, including non-response or worsening symptoms. A small retrospective case series included DME patients receiving only IVR injections. They concluded that OCT biomarkers can aid in predicting the one-year response to IVR Injections in terms of improvement in Best corrected visual acuity (BCVA) and central retinal thickness (CRT) [42]. Results of a recent study investigating the association between visual acuity and structural changes showed that the outcomes of IVR injections are better predicted by decrement of subretinal fluid (SRF) area, and visual acuity gain was best estimated by reduction of the thickness of the photoreceptor layer [43]. However, given the limitations of present studies, these correlations are yet to be reliable for decisions regarding the direction of treatment and re-treatment and must be supported by sufficient evidence.

Many trials have demonstrated a well-tolerated safety profile regarding aVEGF agents with low rates of severe adverse effects. The most common ocular complications associated with IVR injections are conjunctival hemorrhage, vitreous detachment and a rise in IOP. Complications include endophthalmitis, intraocular inflammation, retinal detachment, cataract and glaucoma [44]. 

A study investigating the rates of cerebrovascular and cardiovascular events of ranibizumab using data from previous clinical trials reported risks of arterial thromboembolism, myocardial infarction, stroke, TIA and vascular death, regardless of the applied dose [45, 46]. Further, a recently published population-based cohort study revealed an increased risk of long-term CKD among patients receiving IVR and further supported previous data [47]. 

Ranibizumab lost its patent in 2020 and 2022 in the US and Europe, respectively. This allowed for the development of more cost-effective biosimilars to reduce the treatment burden and increase patient reach [48]. Biosimilars are drugs made from living cells and are similar to the original molecule in terms of safety, efficacy, biological activity, quality characteristics and immunogenicity. The first ranibizumab biosimilar agent was Razumab™, approved in India for treating wAMD, DME, and macular edema following RVO and CNV [49]. Currently, there 2-FDA approved biosimilars. FYB201(Cimerli, ranibizumab-eqrn) and SB11(Byooviz, ranibizumab-nuna) [50, 51]. Other biosimilars are being investigated, with some reaching their primary endpoints for possible approval in the future.

#### Aflibercept

It is a fusion protein consisting of the binding regions of human VEGF Receptors 1 and 2, fused to the Fc region of human IgG, forming what is known as “VEGF trap,” enabling them to bind to VEGF-A with higher affinity than the native receptors and more potently than ranibizumab and bevacizumab, in addition to its distinct ability to bind with VEGF-B and placental growth factor (PIGF) *which differentiates it from other aVEFs* [52, 53]. VISTA and VIVID trials compared the effect of using 2 mg aflibercept every 4 or every 8 weeks with laser photocoagulation on patients with DME. They concluded that significantly improved visual acuity in those receiving aflibercept compared to laser therapy [54]. 

Aflibercept was first approved in the US in 2011 for treatment of nAMD. It has been used widely in ophthalmological conditions for managing DME, macular edema following RVO, Diabetic retinopathy (DR) and most recently as the first pharmacological option for retinopathy of immaturity(ROP) [55]. 

To compare the relative efficacy of aflibercept in treating patients with centrally involved DME in comparison to other commonly used aVEGF (ranibizumab and bevacizumab), aflibercept was found to be more effective than ranibizumab and bevacizumab in patients with poor baseline visual acuity (20/50 or worse). However, there are no clinically significant differences in vision gains in cases of mild vision involvement [56, 57]. 

The standard dose of aflibercept is 2 mg, and intravitreal injections are given each month for the first three injections, then once every two months. (43) Aflibercept has a new FDA-approved high dose of 8 mg for DME, wAMD after showing non-inferior results in BCVA to the standard dosage of 2 mg, without any new safety concerns throughout 48 weeks from phase 2/3 PHOTON trial [58, 59]. This allows for more excellent durability through extended intervals of up to 16 weeks than the previous regimen.

To date, data from trials and real-world studies regarding systemic adverse events of different aVEGF (aflibercept, ranibizumab and bevacizumab) are conflicting. Similar rates of systemic adverse effects have been shown among the three study groups in some incidents [56]. Analysis of some studies revealed an increased risk of stroke among aflibercept and ranibizumab study groups about bevacizumab [60, 61]. 

As stated previously, CKD is a long-term outcome of intravitreal aVEGF injections, alongside the independent risk of developing CKD in diabetic patients. Thus, renal function deterioration unrelated to the DM clinical course necessitates renal biopsy and discontinuation of VEGF injections [62]. Post aVEGF injection, endophthalmitis has generally low incidence rates. However, the list of causative micro-organisms is typically expanding to involve rarely or first-time isolated species like *Morganella morganii* endophthalmitis case reported in a 75-year-old patient after routine intravitreal aflibercept injection [63, 64]. This risk can be minimized when using prefilled syringe preparations. Furthermore, those treated with prefilled aflibercept injections had less chance of developing ocular hypertension with vial-drawn syringes [65]. 

**Fig. 2:**
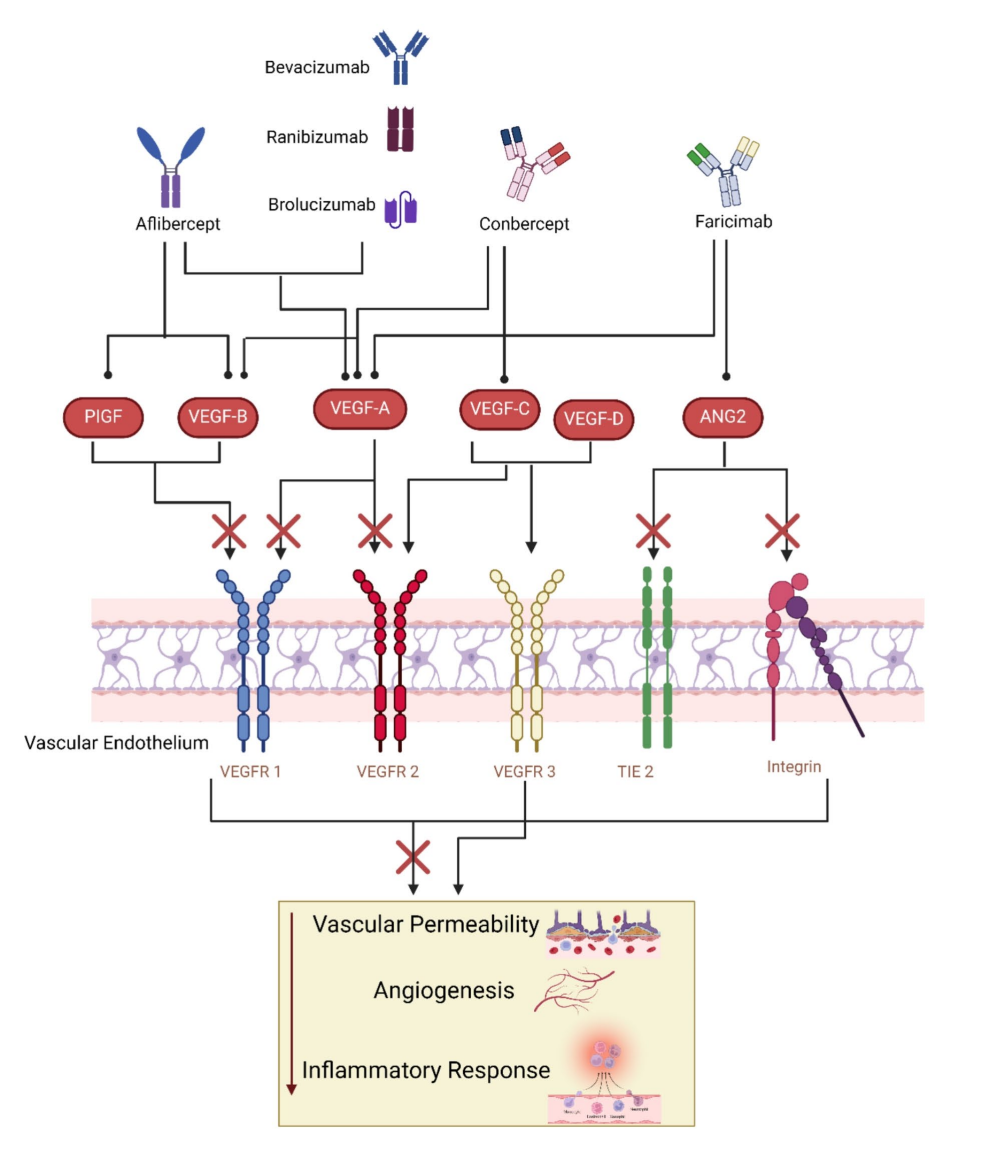
The Role of Anti-VEGF Medications in the Management of Diabetic Macular Edema. VEGF is a signal protein that stimulates the formation of blood vessels. When VEGF is overexpressed, it can lead to the vascular permeability and neovascularization seen in DME. Anti-VEGF drugs work by binding to VEGF, inhibiting its ability to bind to its receptors on the surface of endothelial cells. Different anti-VEGF drugs may have different affinities for the various VEGF receptors, which can influence their efficacy in DME management.

Despite the high efficacy of aflibercept, some patients show poor response to the initial. It is when some ophthalmologists might consider switching to another agent to overcome refractory results. In the case of aflibercept or ranibizumab, promising results were found when switching to faricimab regarding BCVA and extension of injection intervals [66, 67]. Overall, the selection of treatment options and frequency of injections must be adjusted according to patients’ risk factors and specific needs (see Fig. 2).

#### Brolucizumab

It is one of the novel anti-VEGFs that has already been considered as one of the new approaches to DME treatment, which is secondly approved by the FDA in 2022 after primary approval in 2019 for the wet AMD. It is created by a single-chain antibody fragment with a high affinity for suppressing VEGF-A’s ability to bind to VEGF receptors VEGFR1 and VEGFR2 [68, 69]. 

Compared to other anti-VEGFs, brolucizumab has a higher anti-VEGF-molar binding capacity per injection due to its low molecular weight of 26 kDa. Its low molecular weight also gives it a longer half-life and better tissue penetration [69]. An insignificant amount of net liquid volume, 50 µl, can provide 6 mg of intravitreal injection of brolucizumab, 11 times more than aflibercept [70]. 

Moreover, brolucizumab has been determined to be not inferior to aflibercept in the Phase 3 HAWK and HARRIER trials to treat nAMD [71]. Further, The KESTREL and KITE trials were two Phase 3 studies intended to assess the safety and effectiveness of brolucizumab in individuals with DME. Patients with DME were randomly assigned to receive either brolucizumab 3 mg, 6 mg or aflibercept 2 mg in KESTREL or brolucizumab 6 mg or aflibercept 2 mg in KITE. In these 100-week trials, it was found that Brloucizumab 6 mg, dosed up to every 16 weeks, provided clinically notable visual acuity improvements when compared to aflibercept [72]. 

Including the EU and the US FDA, over 40 countries acknowledged brolucizumab for treating DME-related visual impairment [73]. However, when combined with additional factors of intraocular inflammation, using brolucizumab for AMD has been shown to cause retinal vasculitis and retinal vascular occlusion [74]. While various randomized clinical trials show that intraocular inflammation with brolucizumab is not as joint in diabetic macular edema patients as it is in patients with AMD [71, 72], in the course of therapy of DME, only a single case of intraocular inflammation with vascular blockage has been documented [75]. 

#### Bevacizumab

A recombinant humanized monoclonal inhibits VEGF-A’s ability to bind to VEGF receptors VEGFR1 and VEGFR2 [76]. The FDA has approved bevacizumab for treating many malignancies but not for conditions affecting the eyes [77]. Nonetheless, bevacizumab is frequently used intravitreally off-label to wAMD as it has demonstrated encouraging outcomes in enhancing visual acuity and lowering macular edema. Compared to other anti-VEGF drugs like ranibizumab and aflibercept in many randomized clinical trials, comparable results were obtained regarding safety and efficacy. However, Bevacizumab is far less expensive than the other approved medications, making it a desirable choice for individuals and healthcare systems [78–80]. Yet, the FDA has not authorized bevacizumab, which restricts its availability and reimbursement in some circumstances.

Outlook Therapeutics, Inc. has developed (bevacizumab-vikg), a novel ocular formulation of the drug to address these concerns. This formulation aims to produce a standardized and regulated product for intravitreal usage and satisfy the FDA’s standards for a biologics license application (BLA). A critical phase 3 research (NORSE TWO) has shown encouraging results regarding the safety and efficacy of bevacizumab-vikg regarding the mean change in BCVA from baseline to week 52, being non-inferior to ranibizumab. Therefore, by mid-2022, it became the first bevacizumab ophthalmic formulation to receive FDA approval [81, 82]. 

In August 2022, a randomised clinical trial study was published, which involved a total of 312 eyes with DME; 158 eyes had received intravetrial aflibercept 2.0 mg monotherapy and 154 ones received intravetrial bevacizumab 1.25 mg, which later was switched to aflibercept therapy at the beginning of week 12. The two groups had no significant difference in treatment outcomes (visual acuity and retinal central thickness) over two years. These findings suggest that a cost reduction of DME treatment can be achieved since a single dose of aflibercept is about $1.830, while it is about $70 for bevacizumab [83]. Although bevacizumab had proven its safety, the aflibercept monotherapy group developed severe adverse effects in about 52% of the patients with hospitalization compared to the bevacizumab first group with a percentage of 36% for the adverse events and 32% for the hospitalization [83]. 

Further, a recent study concluded that administering vitamin D oral tablets and IVB injections in diabetic macular edema patients may have a significant role in visual improvement. However, it may take several months to be seen [84]. 

#### Faricimab

A new intravitreal injection is being used to treat two of the leading causes of vision loss and impairment: DME and nAMD. It is the first bispecific antibody that targets both angiopoietin-2 (Ang-2) and (VEGF-A) [85, 86]. 

The Tie-2 receptor, which typically encourages vascular stability and maturation, is antagonistic to Ang-2 [87]. In hypoxic environments and in response to VEGF-A stimulation, Ang-2 expression is elevated, which causes Tie-2 deactivation, endothelial dysfunction, increased vascular leakage, inflammation, and angiogenesis [53, 88]. Additionally, Ang-2 increases endothelial cells’ sensitivity to VEGF-A’s actions, generating a positive feedback loop that intensifies vascular damage. By blocking VEGF-A and Ang-2 simultaneously, faricimab breaks this vicious cycle and restores the stability and integrity of the blood vessels. Due to its distinct Cross MAb structure, faricimab can attach to two targets with high affinity since it contains two distinct antigen-binding domains on a single antibody molecule [89, 90]. 

Several phase 3 clinical trials for DME and nAMD have shown faricimab’s efficacy and safety. Faricimab demonstrated no inferiority to aflibercept in increasing best-corrected visual acuity (BCVA) at one year in the YOSEMITE and RHINE trials for DME, with over half of the patients obtaining an extended dose interval of 16 weeks by the end of the study [91]. 

Further, Faricimab also showed no inferiority to aflibercept in improving BCVA at one year in the TENAYA and LUCERNE trials for nAMD, with approximately half of the patients reaching an extended dosage interval of 16 weeks after the research. In both indications, faricimab was usually well tolerated and had a safety profile similar to that of aflibercept [92]. Consequently, a comprehensive systematic review and meta-analysis of 4 RCTs conducted by Li G. et al. in 2023 [93] shows that compared to other anti-VEGF treatments with longer dose intervals, faricimab produces non-inferior or even more significant CST improvement; however, additional long-term follow-up studies are required to reinforce these findings.

Since faricimab targets both VEGF-A and Ang-2, it is the first therapy for DME and nAMD that may potentially lessen the treatment burden while preserving or improving visual results. Health Canada and the Food and Drug Administration (FDA) in the United States have approved faricimab for these indications, and the National Institute for Health and Care Excellence (NICE) in the UK has given it good final draft guidance [85]. 

Further, in a prospective study conducted between November 2022 and August 2023, 28 patients were enrolled to assess the impact of faricimab on the formation and elimination of micro aneurysms (MA) following three months of injections. One eye of the patient received faricimab, with the other serving as a control. In 206, 16, and 103, aneurysms vanished, formed, and were maintained, respectively. Furthermore, the analysis showed that a size reduction was accomplished, resulting in the absence of the medium and small size MA, which, in comparison to the large-sized MA, primarily contribute to leakage [94]. 

#### Conbercept

Conbercept (Lumitin) KH902 is an alternative fusion protein of the VEGF receptor (VEGFR) used by drug developers at Chengdu Kanghong Biotech. It inhibits all variants of VEGF-A, VEGF-B, VEGF-C, and PlGF. It exhibits a solid affinity for VEGF and remains persistent in the vitreous for an extended period due to its prolonged half-life [95, 96]. 

After completing Phase III clinical trials, it was concluded that applying the Conbercept regimen improved BCVA) and (CMT) among patients with DME. Thus, Conbercept is now regarded as a viable option for treating DME. Nevertheless, limited research is available regarding the impact of Conbercept (IVC) in patients with DME [96]. In May 2019, Conbercept acquired marketing approval from the China State Food and Drug Administration (CFDA) to treat patients with DME [97]. 

This recently introduced anti-VEGF medication offers an alternative treatment option for DME, nAMD and macular edema secondary to RVO in China [98–100]. However, Conbercept has not yet been introduced into the markets of other countries. Conbercept has garnered global attention as a promising treatment due to its comparable structure to aflibercept, remarkable safety and efficacy, and more affordable cost [95]. 

Further, Xing P. et al. conducted a retrospective study on 30 patients, analyzing 30 eyes with DME. The study found no improvement in the patient’s eyes before they were on ranibizumab treatment. However, they demonstrated functional improvement upon switching to Conbercept, including enhanced BCVA during at least 6 months of follow-up, suggesting the possibility of reversing functional impairment [101]. 

Moreover, Zhu Z. et al. found that patients with (DME) experienced enhancements in the size of the foveal avascular zone (FAZ) and the density of superficial blood vessels after receiving an intraocular injection of Conbercept. These improvements were particularly significant in patients with diabetic macular ischemia (DMI) at baseline. The injections were administered using the 3 + pro re nata (PRN) principle. Moreover, Conbercept exhibited a beneficial effect on blood flow status in the macula and facilitated the restoration of blood flow in areas where blood flow was previously absent [102]. 

While compared to laser photocoagulation, Liu K. et al. A treatment regimen of 0.5 mg conbercept administered as needed pro re nata (PRN) resulted in significant improvements in both the functional and anatomical outcomes of patients with center involved (DME) versus the results of laser photocoagulation [96] (see Table 1).

**Table 1:** Comparison of current available Anti-VEGFs for DME management.

Drug	Manufacturer	Structure	Mechanism of action	Molecular weight	Year of approval
Brolucizumab	Beovu	Single chain antibody	Recombinant humanized monoclonal antibody VEGF inhibitor that binds to 3 major isoforms of VEGF-A suppressing endothelial cell proliferation, neovascularization, and vascular permeability.	26 kDa	Approved in 2019 for age related macular degeneration. Approved in 2022 for diabetic macula oedema
Bevacizumab	Avastin Alymsys Mvasi Vegzelma Zirabev	Monoclonal antibody	Binds to and neutralizes VEGF preventing its association with nuclear receptors, Flt-1 and KDR.	149 kDa	Not approved for diabetic macula oedema
Faricimab	Vabysmo	Angiopoietin-2 Inhibitor	Inhibits VEGF-A resulting in suppression of endothelial cell proliferation, neovascularization, and vascular permeability. Inhibition of angiopoietin-2 promotes vascular stability and desensitizes blood vessels to effects of VEGF-A.		Approved in 2022 for age related macular degeneration and diabetic macular oedema
Ranibizumab	Byooviz Cimerli Lucentis Susvimo	Antibody fragment	Binds to and inhibit VEGF-A suppressing neovascularization.	48 kDa	Approved in 2006 for Macular Degeneration; Macular Edema; Diabetic Macular Edema; Diabetic Retinopathy; Myopic Choroidal Neovascularization.
Aflibercept	Eylea	Fusion protein	Decoy receptor for VEGF-A and placental growth factor (PlGF) inhibiting binding and activation of endothelial cell receptors thereby suppressing neovascularization.	97-115 kDa	Approved in 2011 for neovascular (wet) age-related macular degeneration, macular edema following retinal vein occlusion, diabetic macular edema, diabetic retinopathy, and retinopathy of prematurity.

### Steroids

Steroids are part of the management plans available for diabetic macular edema, especially for some patients who are unresponsive to other treatment modalities, such as anti-VEGF agents, like pregnant women and patients with recent strokes [103, 104]. About 40% of these patients don’t gain more than 5 letters, nor does their BCVA reach 20/40 [103]. Steroids mainly act by down-regulating the molecules and inflammatory mediators responsible for VEGF synthesis that partakes in the DME pathophysiology [103]. However, they still risk developing complications that render their use less preferred, such as developing cataracts and increased intraocular pressure “IOP.” [104] Steroids administered locally have proven to decrease intraocular inflammation, neovascularization and cellular proliferation [105], so now they are available as sub-tenon triamcinolone acetomide injections, and the newest promising advancement is dexamethasone, and fluocinolone acetonide intravitreal implants, the latter has an effect lasting up to 3 years [7, 104]. However, as effective as literature reviews view them, they still carry an even higher risk of IOP, cataracts and poorer visual acuities than other treatment modalities. Hence, steroids are mostly reserved for non-responders who took 3 to 6 injections of anti-VEGF agents without improvement [7]. 

Current research suggests that combining anti-VEGFs with dexamethasone implant therapy can lead to reversing foveal damage and decreasing the need for injections [104, 106].

#### Injections of triamcinolone acetonide (TA)

Intravitreal injections of TA are mainly used as a safe short-term (< 3month) alternative when the patient is unresponsive to at least 3 doses of bevacizumab or when laser photocoagulation is contraindicated or unhelpful; many clinical studies found that there is a correlation between the dose of TA and treatment clinical outcomes, as higher doses (> 8 mg) appear to have a better chance at improving BCVA and increasing central retinal thickness (CRT). Nonetheless there.

No correlation was found between the dose of TA and the level of IOP elevation [7, 103, 105]. Doses > 13 mg have shown a longer-lasting overall effect and doses > 20 mg have shown a temporary improvement in visual acuity [105]. IOP is the most concerning complication of TA Intravitreal injections (IVTA); it is also the most commonest, affecting about 52% of 104 eyes 6 weeks into treatment. IVTAs also carry the risk of developing endophthalmitis and cataracts [105]. Sub-tenon injections of TA (STTA) seem to have a less effective impact on visual acuity and anatomic responses than their intravitreal counterparts (IVTA); however, these differences seem to be non-existent at the 6-month mark of treatment. Moreover, STTA has led to a higher increase in IOP than IVTA. Due to their short duration and need for multiple injections, they are not used as a monotherapy. They are usually used as an adjunct to anti-VEGF, leading to overall better results and fewer injections [7, 103]. IVTA has been shown to decrease micro-aneurysm number significantly in DME, So its use following anti-VEGF treatment might be beneficial in recurrent or persistent DME [107]. The newest approach being explored is suprachoroidal injections of TA (SCTA); higher doses up to 10 folds can be used with minimal drug infiltrating the anterior segment and lens; thus, a much lower rate of side effects has been observed. IOP increased significantly during the first month but then returned to baseline values at the 3-month mark with topical anti-glaucoma drops, which, in and of itself, is a superior aspect of SCTA over the other modalities of TA administration. Eyes with neurosurgery detachment and disruption of the inner/outer segments had the least favorable prognosis in BVCA improvement [108]. IOP increase appears to always negatively affect optic nerve morphology, even if treated with antihypertensive drugs or in the absence of intra-ocular hypertension [109]. 

#### Intravitreal sustained-release steroid implants

It is a 6-month sustained release implant which is mainly of 2 types; the first type is a non-biodegradable implant made of ethylene vinyl acetate containing a reservoir of the steroid needed such as TA; these implants require surgical implantation into pars plana, and the surgical explanation after drug reservoir is finished, which can lead to extra complications like retinal detachment and vitreous hemorrhage. Biodegradable types are mostly made of degradable polymers like Polylactic acid (PLA) and polyglycolic acid (PGA), so surgical removal is unnecessary. However, biodegradable types are not available with TA but rather with dexamethasone (DEX), rendering them superior to IVTA implants [105, 110]. 

DEX implants have shown a significant improvement in BVCA in treatment-naive patients and a substantial reduction in central macular thickness (CMT) 6 months following treatment.

However, only CMT seemed to improve in treatment-refractory patients, while BVCA remained unchanged significantly [111]. 

DEX implants are superior to IVTA implants because a much lower incidence of ocular hypertension and cataracts was reported using them than with IVTA implants in addition to fewer injections; the average was 1 injection with DEX implants, 1.44 for IVTA implants [110]. However, patients of both groups have improved vision and gained similar results of more than 10 letters along with CMT improvement as there were no significant differences between treatment groups at 6-month follow-up [110, 112]. Fluocinolone acetonide (FA) implants reach their peak efficacy 6 to 11 months post-injection most studies recorded 5 or more letters of improvement. Macular thickness and anatomical fluctuations were stabilized after FA injections, allowing for an enhanced visual acuity up to 3 years follow up [106]. DEX implants also appear to be superior to fluocinolone acetonide (FA) implants due to their lower risk of ocular hypertension and cataracts. However, FA implants tend to last much longer, up to 36 months, thus alleviating some of the treatment and visits burden, reducing the average treatment frequency from once every 3.2 months before FA implant administration to once every 16.7 months post-FA–FA implant. Additionally, about 25.5% of eyes remained free of any adjunctive treatment all through these 36 months [113, 114]. Lipid-based nanocarriers of TA are a novel approach showing promising results in the treatment of DME, Like an increase in foveal thickness, contrast sensitivity and VA. It can be used as an adjuvant or primary therapy and possibly be considered the future replacement of IVTA [105]. Response to extended-release steroid implants in the contralateral eye might occur in some patients, but the effect was mostly insignificant [115]. 

### Systemic therapy

Although diabetic macular edema is treated mostly with laser photocoagulation and intravitreal anti-VEGF and corticosteroid injects, which are local therapies, systemic therapies have been implicated in previous research. These therapies could have a direct local effect on the retina or an indirect effect by affecting systemic parameters associated with diabetic macular edema. Some of the systemic parameters associated with diabetic macular edema are hyperglycemia, dyslipidemia, hypertension and the presence of coronary artery disease and heart failure [116–118]. 

Although inadequate control of diabetes is associated with the worsening of diabetic retinopathy and increased risk of diabetic macular edema, some of the antidiabetic drugs have no adverse effect on the risk and progression of diabetic macular edema. GLP-1 agonists are associated with a neutral effect regarding the risk of diabetic retinopathy and DME, except Semaglutide [119]. Glitazones and insulin are also associated with an increased risk of DME. However, causation should be further investigated as the association between these diseases and DME could be confounded by type 2 diabetes disease increased activity requiring the administration of the drugs [120–122]. However, the adverse effects of glitazones could be explained by increased water retention, and the cessation of these drugs was associated with an improvement in DME after 3 months in a group of patients [123]. Biguanides (Metformin) and SGLT-2 inhibitors were associated with a lower risk of DME. However, there is still a need for randomized controlled trials examining the effects of these drugs on DME progression and risk [124, 125]. Metformin and SGLT-2 inhibitors are suggested to have local effects, with SGLT-2 inhibitors acting on SGLT-2 receptors found in the retina, Metformin affecting the expression and splicing of VEGF [7]. DPP-4 inhibitors were not associated with an increased risk of DME [126]. The effect of the previously mentioned antidiabetic drugs and other antidiabetics should be further investigated [5].

Lipid-lowering drugs were shown to have a positive effect on DME. Statins were shown to be associated with a lower risk of DME, although they do not affect diabetic retinopathy progression [127]. Two randomized controlled trials of fibrates lowered the risk of diabetic retinopathy and DME [7]. In a systematic review, fibrates, but not statins, were shown to reduce the risk of DME, but the evidence is of low certainty [128]. A newer study showed that fibrates were associated with a lower risk of diabetic retinopathy but not DME [129]. Thus, further studies are needed to examine the effects of lipid-lowering drugs on DME risk and progression.

Other systemic therapies potentially managing DME include infliximab, an anti-TNF biologic. Although intravitreal infliximab was ineffective in preclinical trials and trials on a small number of patients and caused adverse effects of uveitis, a randomized controlled trial using systemic infliximab for refractory DME patients showed that infliximab was well tolerated and effective in improving visual acuity [130–132].

Oxygen therapy was shown to be effective for DME, with one randomized controlled trial with a face mask on the oxygen flow rate of 10 L/min leading to improvement in Best-corrected visual acuity (BCVA), optical coherence tomography, fluorescein angiogram and electroretinograms findings [133]. Another more recent prospective study showed improvement in diabetic retinopathy and DME with hyperbaric oxygen therapy [134]. A study done on patients being treated for the diabetic foot with hyperbaric oxygen therapy showed no long-term effect of hyperbaric oxygen therapy on diabetic retinopathy or DME; however, the study aimed to detect any harmful ophthalmological impact of hyperbaric oxygen therapy in diabetic foot patients [135].

### Laser therapy

**Fig. 3:**
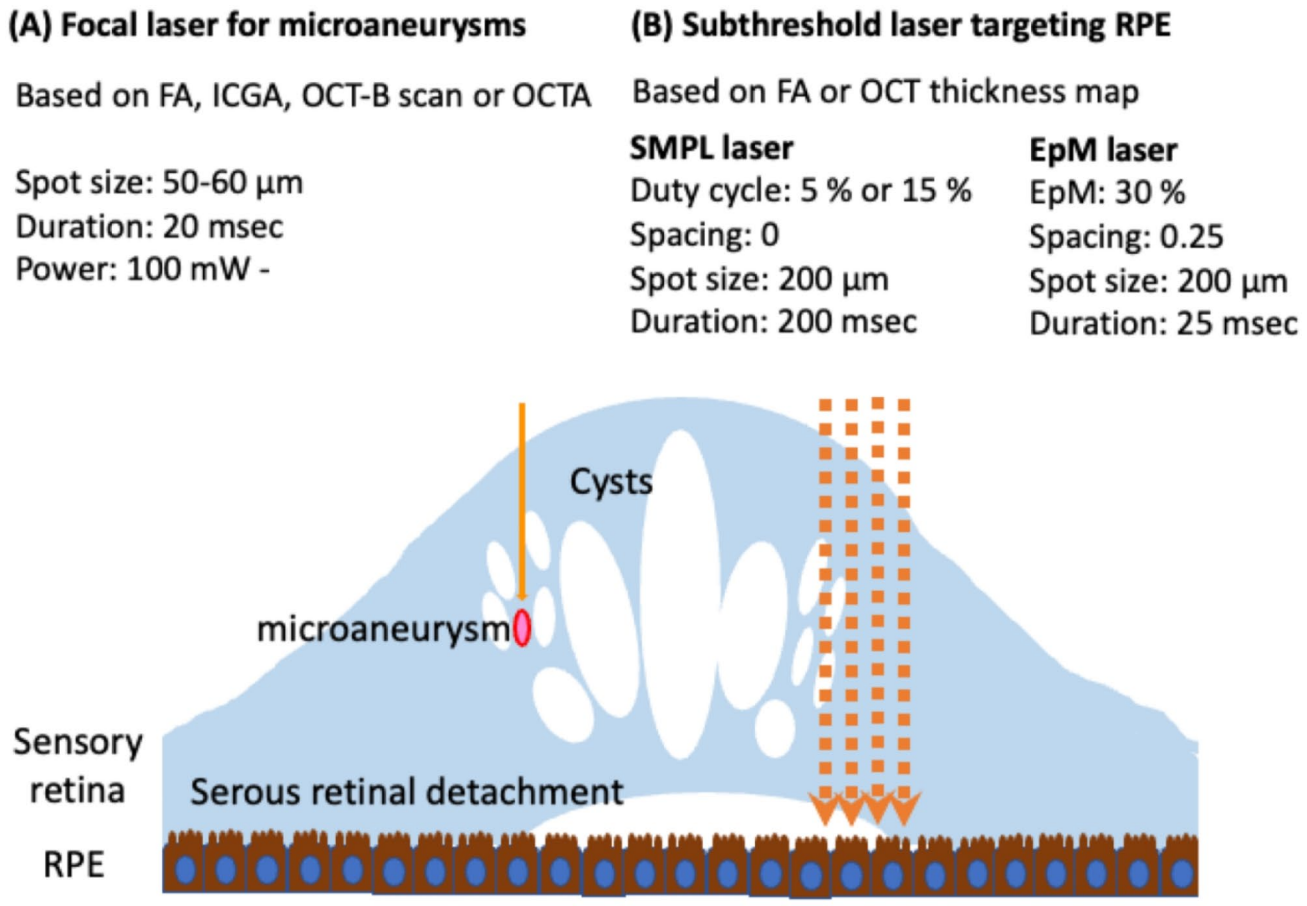
The summary includes the types and parameters of current laser therapies. (**A**) When focusing on microaneurysms with focal laser treatment (indicated by a solid orange arrow), it is advisable to apply the laser for a short duration. To plan the laser treatment, images from fluorescein angiography (FA), indocyanine green angiography (ICGA), optical coherence tomography (OCT) B-scan, or OCT angiography (OCTA) would be utilized. (**B**) Subthreshold laser treatment (indicated by an orange dotted arrow) is aimed at the retinal pigment epithelium (RPE).

Laser therapy targets retinal tissue with light absorbed by the ocular pigment, most commonly in the retina, choroid, melanin, and hemoglobin [136]. The exact mechanism of action is unknown, but one of the theories suggests that light from laser therapy destroys tissue, so less oxygen reaches the target, it gets hypoperfused, and the remaining tissues get hyperperfused. Current laser therapies have many various types and settings (see Fig. 3) [137].

Conventional laser therapy has been used for decades in treating macular edema and has shown clinical significance in reducing the thickening of the retina in two ways (focal or diffuse) [138]. However, burning the area around the retina has an adverse effect, which causes atrophy, scarring, scotoma, and fibrosis [139–141].

After that, the modified laser therapy was invented to reduce the adverse effect because it was less intense, had a smaller spot size, and affected a small area, so it modified the thickness, which contains a small area of the retina and the non-perfusion area [1, 142]. 

Then, **modern laser therapy** evolved, containing both the therapeutic effect of conventional and the less adverse effect of modified laser therapy. the semi-automated pattern scanning retinal photocoagulation system (PASCAL^®^, PAttern SCAn Laser) [143] which contains multiple burns(4–65). A 532-nm wavelength is utilized through a standard slit-lamp system in a specific pattern (grid, arc, etc.). It shows some advantages such as less time, more comfort for the patient, and more accuracy, which avoids making a scar that results in a scotoma. Some studies said that less time of laser reduces the time of burning the retina and more comfort for the patient because less time heating the choroid [144].

Pattern scan laser is highly efficient in treating Diabetic macular edema (DME). It consists of ring and arc patterns with a central foveal exclusion zone. It differs from conventional laser therapy (mentioned above) in that no burn is placed close to the center of the foveal avascular zone. Pattern scan is also used in subthreshold laser [145, 146].

Further, Selective Retinal therapy (SRT) is characterized by 30 pulses ranging from 450 to 800 mJ/cm2 per pulse [158, 159] and a high temperature that targets melanosomes inside retinal pigment epithelium which absorb 50% of green light. There are two types: pulse and continuous wave scanning mode. There are a lot of clinical trials that showed the safety and effectiveness of SRT. For example, a retrospective showed significant effectiveness in the visual accuracy of SRT for 6 months with follow-up up, and there was no adverse effect; however, SRT was not clinically used because it didn’t show any retinal changes and unidentified energy needed for therapeutic damage, so it is challenging to use it clinically [73]. 

Moreover, Subthreshold Diode Micropulse Laser (SDML) was first described for DME in 1997 by Karatza and Fiberg [160]. Since then, several RCTs have shown that SDML is more effective or the same as conventional laser grid therapy. Laursen et al. [161] performed the first RCT in 2004, comparing 810 nm SDML to conventional argon 512 nm laser therapy. This resulted in the same visual acuity, but better thickness SDML was received typically using a longer duration than other studies, which may have made it more successful. In 2009, Figuiera et al. [162] performed a similar study where eyes were treated at baseline and for 4 months if edema persisted. They found no change in visual acuity thickness and contrast sensitivity. Still, they recorded 59% of scars in eyes treated with conventional and 14% with SDML, showing fewer adverse effects. Still, the same effectiveness showed more burn in eyes treated with SDLM than conventional, suggesting that the laser used in SDML led to overtreatment where suggested undertreatment in the traditional group.

**Fig. 4:**
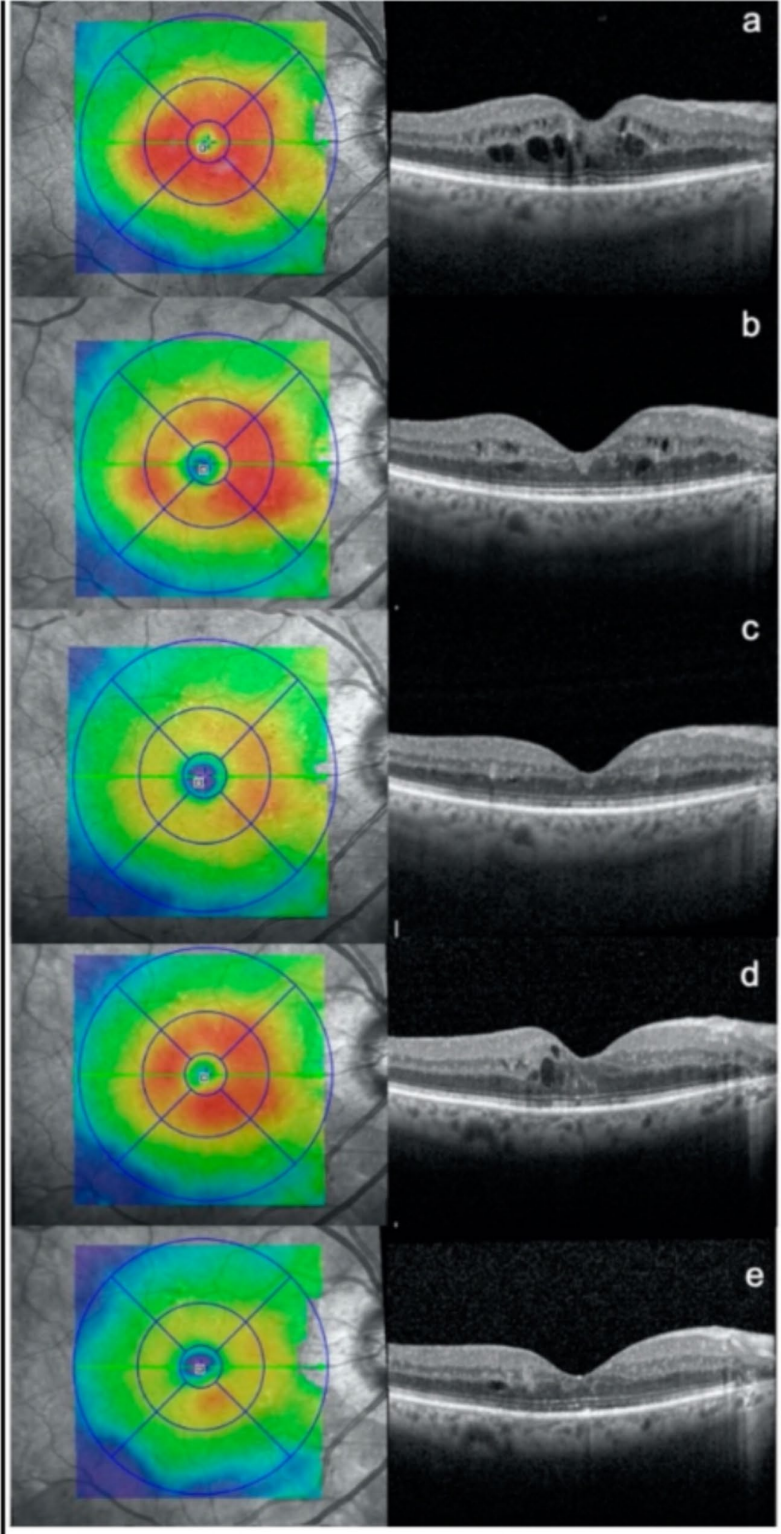
Optical coherence tomography (OCT) scans were taken of a patient with diabetic macular edema at different time points: **(a)** before any treatment, **(b)** 3 months after the first subthreshold micropulse macular laser (SMPL) treatment, **(c)** 3 months after the second SMPL treatment, **(d)** an additional 3 months after the second SMPL treatment, and **(e)** 3 months after the third SMPL treatment.

Another study by Lavinsky in 2011 [147] compared focal laser to normal and high-density SDML and hypothesized that a larger area and less damage effect of SDML is superior to the conventional grid. They were treated at baseline, 6 and 12 months, and they yielded that high-density SDML showed more visual acuity at 12 months, but average density showed no improvement in visual acuity. Macular thickness was the same between all groups, so the result showed that high-density SDML is superior to the other two treatments. Other additional studies compared the previous outcome in addition to electro and psychological and showed no difference, the same as previous studies (see Fig. 4) [148]. In addition, Venkatesh showed fewer signal voids 4/23 in patients treated with SDML than 18/23 in conventional patients, resulting in superior treatment because of the preservation of electro-physical function [147]. Furthermore, Vujosevic et al. [149] have shown an increased significant difference in mean central retinal sensitivity while decreased in conventional. There is a lot more RCT showing the benefaction usage of SDML in the treatment of DME [7, 147]. What makes Subthreshold Diode Micropulse Laser (SDM) unique is that it is a continuous sequence of repetitive short pulses with a long break between them. Hence, the benefit of an extended break is that it can help the retina cool down and avoid burn. It works by stimulating retinal pigment epithelium directly because no damage to the retina occurs, making it work on a large area. SDM showed not only inferiority to the standard but also equivalent to it [150].

### Pars plana vitrectomy

Pars Plana vitrectomy (PPV) is a surgical approach primarily utilized in treating tractional DME in patients with poor response to medical therapy. PPV is a procedure that aims to remove the glycation end products and tractional elements superimposed by diabetes retinopathy, which plays a vital role in the development of macular edema [151, 152]. Results were statistically significant for sub-foveal choroidal thickness (SCT) at 6 months post-operatively and for central macular thickness (CMT) at the 1st, 3rd and 6th month following the operation. VA improved significantly in the 3rd and 6th months after surgery [151]. PPV has also been performed in non-tractional macular edema; despite its controversy, significant anatomical improvement has been reported up to 2 years following surgery. However, visual improvement, although reported to improve in some literature, was not a consistent result. It has been speculated that the mechanism by which PPV improves anatomical and sometimes visual outcomes in DME is increased oxygen and capillary blood delivery to the retina, suppressing VEGF production [152]. The other theory is that PPV removes subtle, unnoticeable tractions and increases the clearance of VEGF [153]. PPV, when paired with the internal limiting membrane (ILM) peeling, didn’t demonstrate any significant differences in the anatomical and functional results in comparison to PPV alone; some researchers also believe that ILM peeling could cause additional unnecessary retinal damage, which has led to multiple reported cases to delayed recovery and improvement in BVCA and CMT when compared to their PPV only counterparts [154]. 

Current studies are exploring the potential benefits and possible outcomes of performing PPV in combination with ILM peeling on treatment-naive eyes with DME instead of eyes with refractory and persistent DME to study the efficacy of PPV without having to deal with the setbacks of irreversible retinal damage due to the condition itself and repeated intravitreal injections [155]. PPV performed for reasons other than diabetic retinopathy has been documented.

in literature to have a protective role against the disease evolution and future complications in.

In addition to slowing down its course [153]. 

Another study investigated the effect of intentionally inducing macular detachment while performing PPV. Results revealed a significantly faster and more significant reduction of CMT at the 1st, 2nd and 4th week, but results were insignificant at the 12th and 24th week of the study. BVCA and morphological outcomes were similar to patients who underwent PPV only [156]. PPV paired with ILM peeling in the presence of dexamethasone intra-vitreal implant did appear to prolong the effect of DEX implants with subsequent prolongation of the time usually needed for macular edema to recur. However, no added benefits or differences were observed in the functional and anatomical results of vitrectomized and non-vitrectomized eyes [157]. 

### Combined therapy

#### Anti-VEGF only vs. anti-VEGF with subthreshold diode micropulse laser

Intravitreal injection of anti-VEGF is a better therapy compared to laser therapy; however, it costs over 100,000 dollars annually and needs the patient to follow the physician’s prescription accurately [73]. Laser therapy shows slower onset but longer acting, costing much less. The newer types of it, like subthreshold, showed fewer side effects, so that’s what makes it better to combine with anti-VEGF therapy and the aim of combining to give less injection with the same outcome. Anti-VEGF plus laser showed the best visual acuity outcome at 6 and 12 months; however, anti-VEGF only showed better macular thickness outcomes at 12 months. Combined, no significant difference in macular thickness was observed at 6 months [158]. 

There are some side effects of monotherapy, such as anxiety, pain after injection, and retinal detachment, so the reason further research needs to be established at the same time with central macular thickness of more than 400 μm anti-VEGF needed because subthreshold therapy works only on less than 400 μm. However, the addition of SDM didn’t improve outcomes, but fewer injections were required, so it cost less and fewer visits were needed [73, 158, 159]. However, further studies are necessary to show the cost-effectiveness and side effects of combining.

#### Intravitreal triamcinolone plus macular laser photocoagulation

Intravitreal Triamcinolone (IVT) is an anti-inflammatory steroid. Randomized clinical trial comparing IVT after MLP or IVT only. It showed a significant decrease in visual acuity more than IVT only, and the combination maintained was reduced 3 months after therapy; however, 40% of the intraocular pressure increased in both groups [150]. However, combined therapy was not superior to IVT alone but had a better effect than laser alone [160].

#### Ranibizumab (Anti-VEGF) plus macular laser photocoagulation

Ranibizumab conducted more improvement in visual acuity than MLP only or MLP with Ranibizumab at 6 months but showed no significant difference at 2 years. Ranibizumab showed a decrease in foveal thickness at 6 months; then, foveal thickness increased in the subsequent follow-up. This is in contrast to Combined therapy, which showed a reduction in thickness during 24 months. The additional MLP showed improvement. Also, fewer injections of Ranibizumab are needed, so it costs much less than Ranibizumab only. Also, combined therapy reduced intraocular pressure and injection, so less drug use [61, 161].

#### Ranibizumab plus dexamethasone

it shows improvement in visual acuity and macular thickness, which significantly reduced at 1 month, and there is a positive correlation after the first injection; however, in a later stage, the outcome is reduced. It is mainly used before irreversible change in the macula because it has a good effect at an early stage. Other treatments showed irreversible changes in the macula because damage had already occurred to the macula [162]. 

#### Intravitreal injection of bevacizumab (IVB) plus focal macular photocoagulation (FMP) therapy

An RCT was conducted at Liaquat University. Between 2019 and 2022, two groups of 130-patient were assigned to receive intravetrial bevacizumab (IVB) alone and IVB plus focal macular photocoagulation (FMP) combination therapy over 3 months with a monthly follow-up for both of the groups. The BCVA was evaluated at the end of the 3 months, and the outcome was that the combined IVB plus FMP treatment showed a superior visual outcome compared to the monotherapy [163]. 

#### Intravitreal injection of Conbercept (IVC) plus macular pulse laser (MPL) therapy

The combined therapy of IVC + laser therapy revealed a better response rate at curing DME due to the role of Laser therapy in repairing the blood-retinal barrier [164]. On the other hand, it may cause damage to capillaries, which would increase thickness and vision loss and reduce the effect over a long time. That’s why we combine it with IVC: because it has a better effect with more extended time, and multiple targets also reduce VEGF and inhibit inflammation, edema, and exudation, thus improving visual acuity. Studies showed that visual acuity and macular thickness were better combined than IVC alone, which proves the result of Qiao et al. [165].

After analysis, there is a lower visual field gray value and visual field defect, and the patient’s light threshold sensitivity to light was a 30° field of view higher. Also, combined therapy showed improvement in the quality of the visual field, and this is due to MPL causing less scaring and less production of coagulation of protein and fewer injections in 6 months, thus reducing drug use [164]. 

#### Intravitreal injection of conbercept (IVC) plus laser photocoagulation therapy

In a comparative study, Zhan H. et al. found that an observation group (conbercept + laser photocoagulation) had a significantly higher total efficacy rate (93.85%) than the control group (laser photocoagulation only) (78.46%). BCVA, retinal thickness, and central macular thickness improved in the combined group. Both groups showed improved levels of vascular endothelial growth factor, interleukin-6, soluble intercellular adhesion molecule-1, and essential fibroblast growth factor after treatment, with the combined group exceeding the control group (*P* < 0.05). Concluding that combined laser photocoagulation and IVR conbercept for DME improve vision and lower intraocular cytokine levels more effectively and safely [166]. 

### Promising therapeutics and futuristic insights for DME management

Although efficacious therapies for Diabetic Macular Edema (DME) are extant, many studies have elucidated that the outcomes in pragmatic settings do not align with the superior results in controlled clinical trials [167]. A contributing factor to this divergence could be attributed to the onerous regimen imposed by extant therapies, which may precipitate less than optimal adherence to treatment protocols, culminating in inadequate management of the condition. Furthermore, some patients may demonstrate partial therapeutic responses despite treatment adherence. These issues underscore the need for therapeutics with enhanced efficacy and prolonged durability. Subsequently, an overview of promising therapeutic advances and insights is discussed.

#### Photobiomodulation therapy

**Fig. 5:**
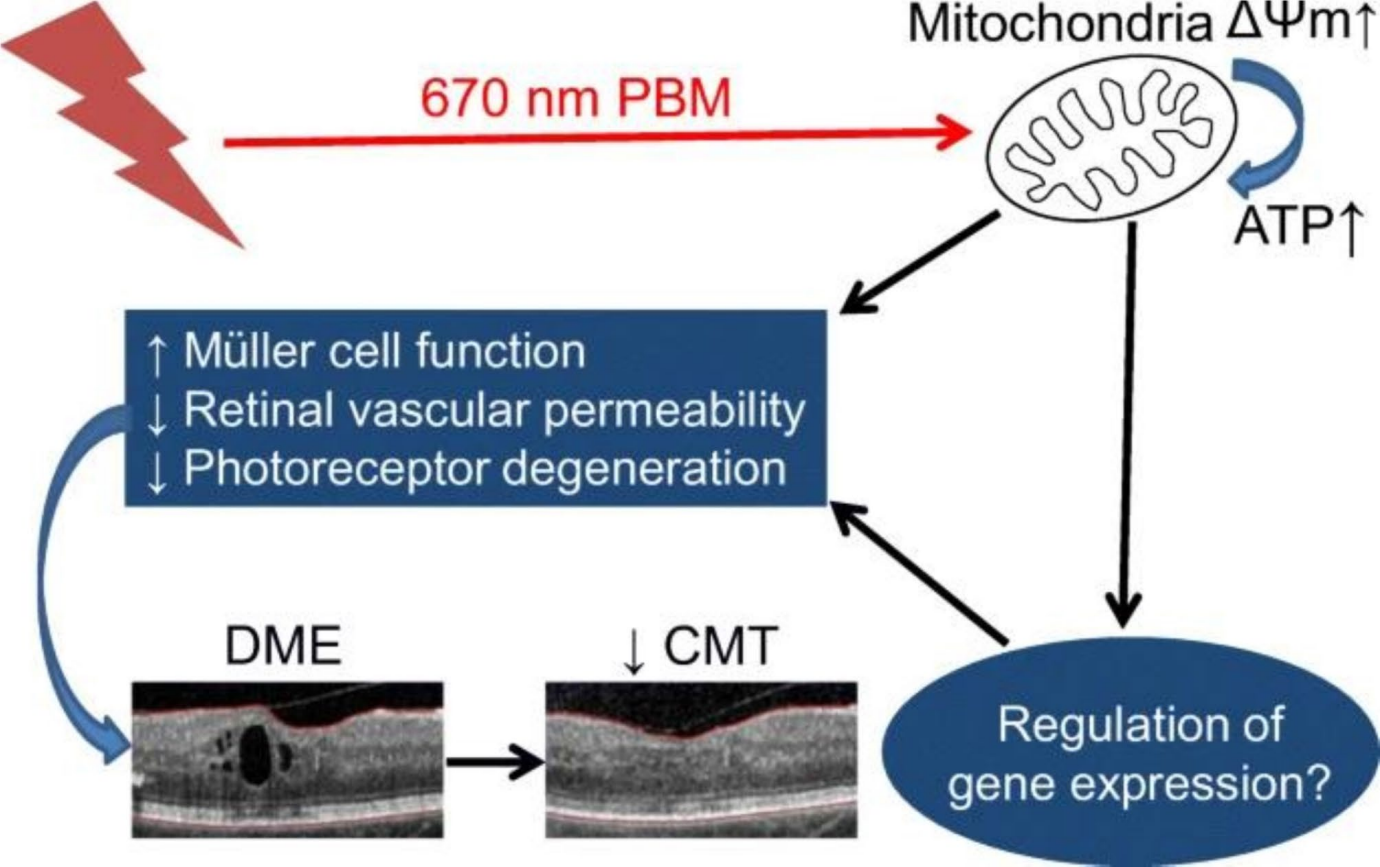
Mechanism of action in laser photobiomodulation.

Low-level light is used in photobiomodulation therapy (PBM), a non-invasive treatment, to promote tissue repair and activate biological processes. PBM functions through a photochemical process in which light is taken up by chromophores found within the cells, like the mitochondria’s cytochrome c oxidase (CCO). This increases mitochondrial activity, ATP synthesis, and reactive oxygen species (ROS) regulation. In addition to their potential to inflict oxidative damage on biological components, reactive oxygen species (ROS) are crucial for signaling and homeostasis. PBM can affect ROS levels differently based on the amount, type, and length of light exposure. By boosting the activity of antioxidant enzymes like superoxide dismutase (SOD), low dosages of PBM can lower ROS levels and catalase by enhancing the repair of oxidative damage [168]. High doses of PBM can raise ROS levels by promoting the synthesis of nitric oxide (NO) and hydrogen peroxide (H2O2). These molecules can then activate different signaling pathways, including nuclear factor-kappa B (NF-κB) and mitogen-activated protein kinase (MAPK), and cause cellular responses, including inflammation, proliferation, and apoptosis [169]. DME is a common consequence of diabetes that results in swelling and fluid leaking in the central retina, ultimately leading to vision loss. PBM has been suggested as a viable therapy for DME. Intravitreal injections of corticosteroids or anti-VEGF medications are typically used to treat DME. Still, these treatments have several drawbacks, including high cost, frequent injection requirements, and infection risk. PBM works by increasing mitochondrial activity, decreasing oxidative stress, and regulating inflammation in the retinal cells to improve retinal function and reduce vascular leakage. Numerous preclinical and clinical investigations have examined PBM’s impact on DME. PBM administered via a retinal laser or light-emitting diode (LED) device has been demonstrated in animal models to shield Müller cells and photoreceptors from harm., increase mitochondrial membrane potential, and decrease retinal vascular leakage induced by Müller cell disruption [170, 171] (see Fig. 5) [170].

**Fig. 6:**
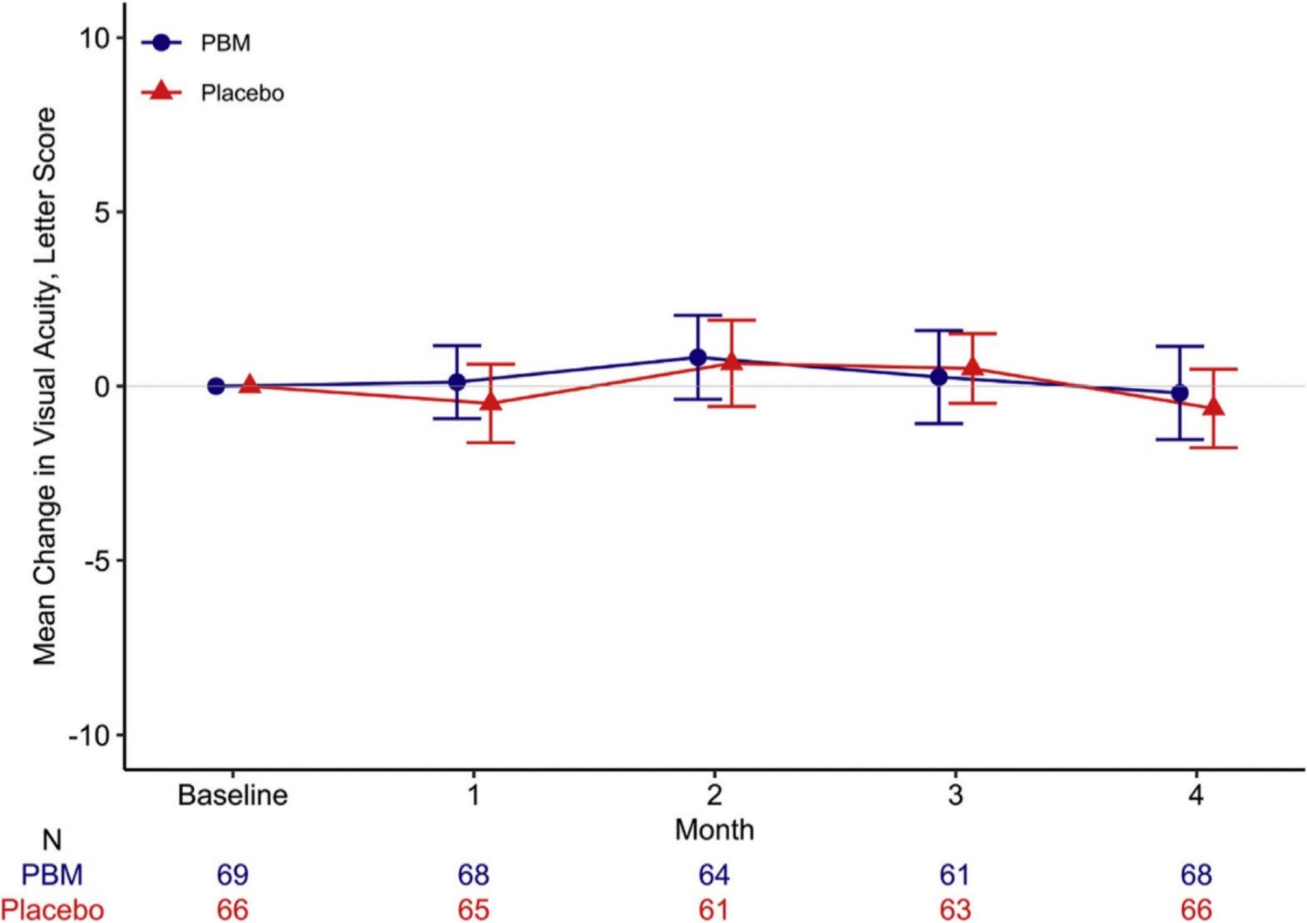
The average difference in visual clarity (with 95% confidence intervals) between the start and 4 months into a preliminary investigation of photobiomodulation (PBM) treatment for diabetic macular edema (Protocol AE).

In human trials, PBM delivered by an LED eye patch or a retinal laser has been tested in patients with center-involved DME and good visual acuity. In a phase 2 randomized clinical trial by Kim et al. [187], 135 patients were compared with PBM eye patches and placebos, and at 4 months, there was no discernible change in either central subfield thickness (CST) or visual acuity (VA) (see Fig. 6). PBM retinal laser was used in 21 patients in a phase 2a open-label dose-escalation trial by Shen et al. The results showed a substantial decrease in CST at 2 and 6 months but no significant change in VA [172]. From April 2019 to February 2020, a study randomly assigned 135 adults to PBM (*n* = 69) or placebo (*n* = 66). The median age of the participants was 62 years, with 37% being women and 82% identifying as White. The PBM group showed a median device compliance of 92%, while the placebo group had 95% compliance. Over the 4 months, OCT CST (central subfield thickness) increased by a mean (SD) of 13 (53) µm in PBM eyes and 15 (57) µm in placebo eyes, resulting in a mean difference (95% confidence interval [CI]) of − 2 (− 20 to 16) µm (*P* = 0.84).CI-DME (center-involved diabetic macular edema) was identified in 90% of PBM eyes and 86% of placebo eyes at the 4-month mark, based on DRCR Retina Network sex- and machine-based thresholds. The adjusted odds ratio for CI-DME was 1.30 (95% CI: 0.44–3.83; *P* = 0.63). Visual acuity (VA) experienced a mean (SD) decrease of − 0.2 (5.5) letters in the PBM group and − 0.6 (4.6) letters in the placebo group, with a difference (95% CI) of 0.4 (− 1.3 to 2.0) letters (*P* = 0.64). Regarding safety, there were 8 adverse events potentially related to the PBM device and 2 to the placebo device. Fortunately, none of these events were classified as severe [173]. The findings of these investigations indicate that PBM may help individuals with DME by lowering macular edema but not by improving eyesight. However, the data is restricted by the lack of defined outcome measures, short follow-up period, heterogeneity of PBM parameters, and small sample size. Larger, more thorough trials are required to confirm PBM’s safety and effectiveness for DME and to improve the treatment plan.

#### Tarcocimab tedromer (KSI-301, kodiak sciences)

A novel intravitreal anti-VEGF, is being evaluated and developed for treating diabetic macular edema. Tarcocimab tedromer has been developed as a novel approach to VEGF inhibition by acting as an antibody biopolymer conjugate. The molecular size had been enlarged to 950 kDa by attaching the IgG1 VEGF antibody portion to a large biopolymer to prolong the intravitreal residence time [103, 174]. The ocular tissue half-life for both retina and choroid for Tarcocimab tedromer is more than 10.5 days and 12.5 days, respectively, as has been shown in rabbit models [175, 176]. The GLEAM and GLIMMER studies are identically designed, randomized, double-masked, active comparator-controlled studies for evaluating the efficacy, durability and safety of tarcocimab tedromer in 460 and 457 treatment-naïve subjects with diabetic macular edema, respectively. However, high proportions of patients on meaningfully longer treatment intervals were observed with tarcocimab tedromer, with half of the patients on every 24-week dosing at the primary endpoint. The primary efficacy endpoints of the GLEAM and GLIMMER studies were to show the non-inferiority of tarcocimab tedromer (dosed every 8 to 24 weeks after 3 months of loading doses) in comparison to aflibercept (given every 8 weeks after 5 months of loading doses) for visual acuity gains. The two studies did not meet these primary efficacy endpoints; However, an increase in adverse effects was observed during the studies in which cases of cataracts were reported using tarcocimab tedromer. Thus, Kodiak’s initial evaluation suggests the failure of both studies and a decision to discontinue further development of Tarcocimab tedromer (KSI-301) was made [177]. 

#### OPT-302 (sozinibercept, opthea)

Represents an investigational soluble fusion protein that targets two growth factors, VEGF-C and VEGF-D, diverging from conventional anti-VEGF agents which solely inhibit VEGF-A and VEGF-B. The scientific consensus posits that adjunctive therapy with OPT-302 in conjunction with established anti-VEGF treatments may confer augmented and sustained therapeutic outcomes for individuals afflicted with Diabetic Macular Edema (DME) relative to monotherapy [178]. In a cohort of DME patients previously administered aflibercept, the phase 2a trials conducted by Opthea revealed that the visual acuity in subjects receiving a combination regimen of OPT-302 and aflibercept exhibited an average enhancement of 6.6 letters at the 12-week mark, in contrast to a 3.4-letter improvement observed in the monotherapy group [179]. Moreover, 27% of the combination therapy group experienced a visual acuity gain of 10 letters or more, a significant contrast to the absence of such improvement in the aflibercept-alone cohort. Notably, OPT-302 was associated with minimal adverse effects in these initial studies. Presently, OPT-302 is under scrutiny in the phase 3 SHORE and COAST trials, targeting the management of wet age-related macular degeneration (AMD) [180]. 

#### MYL-1701P (momenta pharmaceuticals and mylan)

A novel drug that inhibits the activity of two proteins involved in abnormal blood vessel growth: placental growth factor (PEGF) and VEGF-A. It is a potential substitute for Eylea, a current treatment for diabetic macular edema (DME) [78]. In October 2021, a research team conducted a phase 3 trial to compare the safety and effectiveness of MYL-1701P and Eylea in 324 patients with DME. The patients received the drug until week 48 of the study [78].

#### CT-P42 (celltrion)

A recently manufactured drug administered via intravitreal injection to treat DME and AMD. It effectively inhibits the activity of PGF, VEGF-A, and VEGF-B. The drug showed good tolerability in a trial involving about 200 patients with DME, according to a study presented at the annual meeting of the American Society of Retina Specialists (ASRS). So far, this drug has yet to be approved by the FDA, despite its high expectations, especially after the completion of the phase 3 trial in February 2021, which evaluated its efficacy and safety compared with Eylea [181].

#### IBI_324 (innovent biologics)

A drug that targets Ang 2 (Angiopoietin-2) and VEGF-A and inhibits both for patients with DME. The drug has two main strategies to inhibit the formation of abnormal neovascularization: The N-terminal blocks the signaling pathways mediated by VEGF-A, and the C-terminal prevents the binding of Ang 2 to the Tie 2 receptor. The manufacturer presented the key points about the drug, including the phase 1 trial’s evaluation, which covered the efficacy, safety and tolerability. No undesirable ocular side effects occurred even when the maximum dose of 4 mg was administered in the trial [182, 183]. In the SAD phase, a notable response was observed in both BCVA and CST up to day 42 after the dose of each treatment group. In the MAD phase, after one month of the third dose, a clear improvement in the baseline of BCVA was observed by 6.7 ± 5.4 letters in the group with the 2 mg dose and 7.7 ± 4.7 letters in the group with the 4 mg dose of the drug [182, 183]. 

#### OCS-01 eye drop (oculis SA)

A topical corticosteroid with a potential alternative to the more invasive intravitreal steroid injections. In a phase 2 clinical trial, subjects administered with OCS-01 manifested a substantial diminution in central macular thickness, recording a mean reduction of − 53.6 μm, as opposed to a − 16.8 μm reduction in the placebo cohort, over 12 weeks [184]. Despite these promising results, OCS-01 did not correlate with a marked enhancement in visual acuity (VA). The compound was predominantly well-received, although there was a notable incidence of elevated IOP among certain participants. Meanwhile, a phase 3 trial DIAMOND study is ongoing. Preliminary findings indicate a significant improvement in visual acuity for DME patients treated with OCS-01, with gains of + 7.2 letters at 6 and 12 weeks, compared to + 3.1 letters in the placebo group [185]. 

#### Genentech’s port delivery system (PDS) with ranibizumab

A surgically implanted device already approved for treating wet AMD. It administers an intravitreal dose of a concentrated ranibizumab formulation (100 mg/mL). The primary endpoint was met in the PAGODA phase 3 trial, which randomized 634 DME patients to receive monthly ranibizumab or PDS with refill exchanges every six months. The best-corrected visual acuity averaged over weeks 60 and 64 was comparable between the two arms. Furthermore, PDS was well tolerated; over 95% of patients did not need any additional treatments during the two refill cycles that were required in between required refill exchanges [186]. Notably, PDS was voluntarily recalled in October 2022 because of safety concerns about septum dislodgment; however, it is now back on the market [187]. 

#### Gene therapy

A single treatment could remove the need for painful, repetitive, and frequent injections, making gene therapy an appealing option for treating retinal diseases. The modified adeno-associated vector 2–2-associated intravitreal gene therapy known as ADVM-022 (Adverum Biotechnologies) encodes for a chemical akin to aflibercept, which was being investigated for DME in the INFINITY phase 2 trial. The outcomes demonstrated anatomical and optical efficacy and a noteworthy decrease in treatment load compared to pretreatment. However, safety concerns—specifically, intraocular inflammation and hypotony, which caused considerable vision loss in some—led to the suspension of the trial [188, 189]. 

Another intriguing intravitreal gene treatment is called 4D-150 (4D Molecular Therapeutics). It delivers a transgene encoding for aflibercept and a VEGF-C RNAi, efficiently blocking VEGF-A, VEGF-B, VEGF-C, and PlGF (placental growth factor) through a proprietary R100 vector. In treating wet AMD, the phase 1 PRISM study demonstrated a favorable safety profile for 4D-150; the phase 2 SPECTRA trial currently enrolls patients with DME [190]. 

#### Tyrosine kinase inhibitors (TKIs)

TKIs block angiogenesis-related pathways intracellularly, potentially providing a novel means of treating DME. An oral version of the TKI imatinib [191], YD-312 (YD Global Life Sciences Co, Ltd.), was first created to treat chronic myeloid leukemia. When YD-312 was compared to placebo therapy, phase 2a testing revealed a significant improvement in baseline visual acuity. Phase 2b testing is what [192] YD Global Life Sciences Co., Ltd. intends to move on to. Another TKI, EYP-1901 (EyePoint Pharmaceuticals), delivers vorolanib via a bioerodible delivery system that may provide up to a six-month shelf life. There were no noteworthy safety concerns during EYP-1901 treatment, according to the phase 1 DAVIO trial [193]. A study that examined the care of individuals with DME was initiated in early 2024.

#### Kallikrein-kinin (KK) inhibitors

Studies have shown that kallikrein levels are approximately 11 times higher in patients with DME compared with healthy subjects, suggesting that blockage of kallikrein may slow DME progression, specifically by limiting retinal permeability [73, 194]. THR-149 (Oxurion NV, Leuven, Belgium) is a bicyclic peptide inhibitor of human plasma kallikrein. A phase 1, open-label, multicenter dose-escalation study with 3-month follow-up evaluated the safety and efficacy of a single intravitreal injection of THR-149 at three dose levels (5, 22, 130 µg) in 12 DME patients [195, 196]. All subjects completed the study, and no serious adverse events were recorded. By the third month, there was a mean change from Baseline BCVA of + 7.5 letters on Day 14 and + 6.4 letters. The average CST change from baseline at month 3 was + 30.0 μm.

In early clinical trials, therapies that modulate the PK pathway for treating DME yielded mixed outcomes. Further studies examining the efficacy of PK inhibitors, compared to and combined with the standard of care, are warranted. Oral PK inhibitors are also being developed for DME, which, if practical, could decrease physician and patient burden [197]. Additional research will aid in determining whether oral PK inhibitors could play a role in DME.

RZ402 (Rezolute, Inc.) is an oral KI; phase 1b results demonstrated that RZ402 had high bioavailability, significant dose-dependent activity, and minimal safety concerns [198]. A phase 2 trial is currently enrolling and will compare RZ402 with placebo therapy.

#### Senolytic therapy

Senolytic therapy is thought to have a significant role in developing retinal microvascular instability, which is connected to the pathophysiology of DME and DR. Senolytic compounds are specifically made to kill senescent cells [199, 200]. An experimental medication called UBX1325 (Unity Biotechnology) inhibits BCl-xL and encourages apoptosis in senescent cells. In a first-phase study involving eight DME patients receiving UBX1325, after 24 weeks, 62.5% of patients had improved their VA by five letters, and 50% had improved it by ten letters [201]. These results were partially supported by the phase 2 BEHOLD study, which found that after 48 weeks, patients treated with UBX1325 compared to sham injections had non-statistically significant improvements in visual acuity (+ 6.6 letters vs. +0.6 letters, *P* = 0.120) [202]. Phase 2b of the ASPIRE study will be initiated by the end of 2023. Larger RCTs are required to verify the previous results regarding UBX1325 [200] (see Table 2).

**Table 2:** Futuristic agents undergoing investigative RCTs for DME management.

Product name of drug	Manufacturer name	Drug mechanism	Current status
OPT-302 (sozinibercept)	Opthea Ltd., Australia.	Inhibits VEGF-C and VEGF-D activity.	Undergoing phase 2a for DME and phase 3 trial for wet AMD.
PDS with ranibizumab	Genentech, Inc., United States.	Delivers a concentrated formulation of ranibizumab intravitreally.	In phase 3 trial for DME, Approved for wet AMD.
OCS-01	Oculis SA, Switzerland.	Topical corticosteroid for patients with DME.	Undergoing in phase 3 trial called DIAMOND for DME.
YD-312	YD Global Life Sciences Co, Ltd., South Korea.	Oral tyrosine kinase inhibitor that inhibits pathways involved in angiogenesis.	Completed phase 2a testing, planning to progress to phase 2b testing.
EYP-1901	EyePoint Pharmaceuticals, Inc., United States.	Bioerodible delivery system that delivers vorolanib.	Completed phase 1 trial, scheduled to begin a trial for DME in early 2024.
THR-149	Oxurion, Belgium.	Kallikrein inhibitor.	Phase 1 study completed, data forthcoming.
RZ402	Rezolute, Inc., United States.	Oral kallikrein inhibitor.	Phase 2 trial enrolling.
UBX1325	Unity Biotechnology, Inc., United States.	Senolytic compound that inhibits BCI-xl and promotes apoptosis in senescent cells.	Phase 2b ASPIRE study expected to begin in early 2024.
ADVM-022	Adverum Biotechnologies, Inc., United States.	Intravitreal gene therapy that encodes for a molecule similar to aflibercept.	Phase 2 trial halted due to safety concerns.
4D-150	4D Molecular Therapeutics, Inc., United States.	Blockade of VEGF-A, VEGF-B, VEGF-C, and PIGF.	Phase 2 SPECTRA trial enrolling.
MYL-1701P	Momenta Pharmaceuticals and Mylan, United States.	Inhibits VEGF-A and placental growth factor activity.	Undergoing in phase 3 trial for DME.
CT-P42	Celltrion, South Korea.	Inhibits PGF, VEGF-A, and VEGF-B.	Not approved by the FDA yet.
IBI-324	Innovent Biologics, Inc., China.	Inhibits VEFF-A and Ang-2.	Undergoing in phase 1 trial.

## Conclusion

DME is considered among the leading causes of vision loss worldwide. Current guidelines show that DME is primarily treated with anti-VEGF therapy, while other alternative or synergistic therapies are available, including intravitreal steroids, laser therapy, vitrectomy and systemic therapeutic approaches. Although anti-VEGFs show significant benefits for millions of patients globally. It carries a high treatment burden, adverse effects and inadequate response in some DME patients. Emerging therapies including newly discovered Anti-VEGFs, biosimilars, PDS, photobiomodulation therapy (PBM), gene therapy, Tyrosine kinase inhibitors (TKIs), Kallikrein-Kinin (KK) Inhibitors and Senolytic Therapy may offer new mechanisms of action that allow for better efficacy, durability, and safety, their continued advancement is vital to further refining outcomes for the growing number of patients affected by DME. Subsequent large clinical trials ought to yield adequate proof to support these innovative treatments and therapeutic modalities.

## Data Availability

No datasets were generated or analysed during the current study.

## References

[1] Kim EJ, Lin WV, Rodriguez SM, Chen A, Loya A, Weng CY. Treatment of Diabetic Macular Edema. Curr Diab Rep. Sep. 2019;19(9):68. 10.1007/s11892-019-1188-4. 10.1007/s11892-019-1188-4 31359157

[2] Jain R, Daigavane S. Intravitreal OZURDEX vs. Intravitreal Bevacizumab for Diabetic Macular Edema: a Comprehensive Review. Cureus Mar. 2024. 10.7759/cureus.56796. 10.7759/cureus.56796 PMC11036026 38654796

[3] Yau JWY, et al. Global prevalence and major risk factors of Diabetic Retinopathy. Diabetes Care. Mar. 2012;35(3):556–64. 10.2337/dc11-1909. 10.2337/dc11-1909 PMC3322721 22301125

[4] Romero-Aroca P, Baget-Bernaldiz M, Pareja-Rios A, Lopez-Galvez M, Navarro-Gil R, Verges R. Diabetic Macular Edema Pathophysiology: vasogenic versus inflammatory. J Diabetes Res. 2016;2016:1–17. 10.1155/2016/2156273. 10.1155/2016/2156273 PMC5059543 27761468

[5] Merante D, Menchini F, Truitt KE, Bandello FM. Diabetic Macular Edema. Drug Saf. Aug. 2010;33(8):643–52. 10.2165/11538340-000000000-00000. 10.2165/11538340-000000000-00000 20635822

[6] Bandello F et al. Diabetic Macular Edema, 2017, pp. 102–38. 10.1159/000455277 10.1159/000455277 28351052

[7] Tatsumi T. Current treatments for Diabetic Macular Edema. Int J Mol Sci. May 2023;24(11):9591. 10.3390/ijms24119591. 10.3390/ijms24119591 PMC10253534 37298544

[8] CDC, CDC – 2007 National Diabetes Fact Sheet. 2007. [Online]. Available: http://www.cdc.gov/diabetes/pubs/factsheet07.htm

[9] Viswanath K, McGavin DDM. Diabetic retinopathy: clinical findings and management., *Community eye Heal.*, vol. 16, no. 46, pp. 21–4, 2003, [Online]. Available: http://www.ncbi.nlm.nih.gov/pubmed/17491851 PMC1705856 17491851

[10] Brownlee M. Biochemistry and molecular cell biology of diabetic complications. Nature. May 2001;414(6865):813–20. 10.1038/414813a. 10.1038/414813a 11742414

[11] Wong TY, Cheung CMG, Larsen M, Sharma S, Simó R. Diabetic retinopathy. Nat Rev Dis Prim. May 2016;2:16012. 10.1038/nrdp.2016.12. 10.1038/nrdp.2016.12 27159554

[12] Cai X, McGinnis JF. Diabetic retinopathy: animal models, therapies, and perspectives., *J. Diabetes Res.*, vol. 2016, p. 3789217, May 2016, 10.1155/2016/3789217 10.1155/2016/3789217 PMC4736804 26881246

[13] Gundogan FC, Yolcu U, Akay F, Ilhan A, Ozge G, Uzun S. Diabetic macular edema. Pakistan J Med Sci. May 2016;32(2):505–10. 10.12669/pjms.322.8496. 10.12669/pjms.322.8496 PMC4859054 27182271

[14] Cheung N, Mitchell P, Wong TY. Diabetic retinopathy. Lancet. May 2010;376(9735):124–36. 10.1016/S0140-6736(09)62124-3. 10.1016/S0140-6736(09)62124-3 20580421

[15] Vivanco-Rojas O, López-Letayf S, Londoño-Angarita V, Magaña-Guerrero FS, Buentello-Volante B, Garfias Y. Risk factors for Diabetic Retinopathy in Latin America (Mexico) and the World: a systematic review and Meta-analysis. J Clin Med. May 2023;12(20). 10.3390/jcm12206583. 10.3390/jcm12206583 PMC10607496 37892721

[16] Schreur V, et al. Risk factors for development and progression of diabetic retinopathy in Dutch patients with type 1 diabetes mellitus. Acta Ophthalmol. Aug. 2018;96(5):459–64. 10.1111/aos.13815. 10.1111/aos.13815 PMC6174939 30188024

[17] Shi Y, Fan X, Zhang K, Ma Y. Association of the endothelial nitric oxide synthase (eNOS) 4a/b polymorphism with the risk of incident diabetic retinopathy in patients with type 2 diabetes mellitus: a systematic review and updated meta-analysis. Ann Med. May 2023;55(1):2226908. 10.1080/07853890.2023.2226908. 10.1080/07853890.2023.2226908 PMC10291908 37353997

[18] Chitturi SP, et al. REal-world treatment outcomes after delayed intRavitreal therapy in center-involving diabetic macular edema – RETORT study. Int J Retin Vitr. Mar. 2023;9(1):22. 10.1186/s40942-023-00463-y. 10.1186/s40942-023-00463-y PMC10061690 36998064

[19] Sen S, Ramasamy K, Sivaprasad S. Indicators of Visual Prognosis in Diabetic Macular Oedema. J Pers Med. May 2021;11(6):449. 10.3390/jpm11060449. 10.3390/jpm11060449 PMC8224579 34067442

[20] Teo ZL, et al. Global prevalence of Diabetic Retinopathy and Projection of Burden through 2045: systematic review and Meta-analysis. Ophthalmology. May 2021;128(11):1580–91. 10.1016/j.ophtha.2021.04.027. 10.1016/j.ophtha.2021.04.027 33940045

[21] Gangnon RE, et al. A Severity Scale for Diabetic Macular Edema Developed from ETDRS Data. Investig Opthalmology Vis Sci. Nov. 2008;49(11):5041. 10.1167/iovs.08-2231. 10.1167/iovs.08-2231 PMC3028439 18539929

[22] Kao C-C, Hsieh H-M, Lee DY, Hsieh K-P, Sheu S-J. Importance of medication adherence in treatment needed diabetic retinopathy. Sci Rep. Sep. 2021;11(1):19100. 10.1038/s41598-021-98488-6. 10.1038/s41598-021-98488-6 PMC8476599 34580364

[23] Chen L, et al. The learning curve of unilateral Biportal Endoscopic (UBE) spinal surgery by CUSUM Analysis. Front Surg. Apr. 2022;9. 10.3389/fsurg.2022.873691. 10.3389/fsurg.2022.873691 PMC9099005 35574554

[24] Zhang J, et al. Diabetic Macular Edema: current understanding, Molecular mechanisms and therapeutic implications. Cells. Oct. 2022;11(21):3362. 10.3390/cells11213362. 10.3390/cells11213362 PMC9655436 36359761

[25] Yan W, McGuinness M, Chakrabarti R, Fotis K, Finger RP. Comparison of photographic screening methods for Diabetic Retinopathy - A Meta-analysis. Ophthalmic Epidemiol. May 2023;30(3):221–9. 10.1080/09286586.2022.2065311. 10.1080/09286586.2022.2065311 35599625

[26] Aptel F, Denis P, Rouberol F, Thivolet C. Screening of diabetic retinopathy: Effect of field number and mydriasis on sensitivity and specificity of digital fundus photography, *Diabetes Metab.*, vol. 34, no. 3, pp. 290–293, Jun. 2008, 10.1016/j.diabet.2007.12.007 10.1016/j.diabet.2007.12.007 18406188

[27] Xiao Y, et al. Assessment of early diabetic retinopathy severity using ultra-widefield Clarus versus conventional five-field and ultra-widefield Optos fundus imaging. Sci Rep. May 2023;13(1):17131. 10.1038/s41598-023-43947-5. 10.1038/s41598-023-43947-5 PMC10564714 37816867

[28] Kárason KT, Vo D, Grauslund J, Rasmussen ML. Comparison of different methods of retinal imaging for the screening of diabetic retinopathy: a systematic review. Acta Ophthalmol. May 2022;100(2):127–35. 10.1111/aos.14767. 10.1111/aos.14767 33529402

[29] Ullah W, et al. Cost-effectiveness and diagnostic accuracy of telemedicine in macular disease and diabetic retinopathy: a systematic review and meta-analysis. Med (Baltim). May 2020;99(25):e20306. 10.1097/MD.0000000000020306. 10.1097/MD.0000000000020306 PMC7310976 32569163

[30] Prayogo ME, et al. Accuracy of Low-Cost, smartphone-based retinal photography for Diabetic Retinopathy Screening: a systematic review. Clin Ophthalmol. May 2023;17:2459–70. 10.2147/OPTH.S416422. 10.2147/OPTH.S416422 PMC10443682 37614846

[31] Goh JKH, Cheung CY, Sim SS, Tan PC, Tan GSW, Wong TY. Retinal imaging techniques for diabetic retinopathy screening. J Diabetes Sci Technol. May 2016;10(2):282–94. 10.1177/1932296816629491. 10.1177/1932296816629491 PMC4773981 26830491

[32] Cui Y, et al. Comparison of widefield swept-source optical coherence tomography angiography with ultra-widefield colour fundus photography and fluorescein angiography for detection of lesions in diabetic retinopathy. Br J Ophthalmol. May 2021;105(4):577–81. 10.1136/bjophthalmol-2020-316245. 10.1136/bjophthalmol-2020-316245 PMC9092310 32591347

[33] Nguyen QD, et al. Ranibizumab for Diabetic Macular Edema. Ophthalmology. Apr. 2012;119(4):789–801. 10.1016/j.ophtha.2011.12.039. 10.1016/j.ophtha.2011.12.039 22330964

[34] Elman MJ, et al. Randomized Trial evaluating Ranibizumab plus prompt or deferred laser or triamcinolone plus prompt laser for Diabetic Macular Edema. Ophthalmology. 2010;117(6):1064–77. 10.1016/j.ophtha.2010.02.031. e35, Jun. 20427088 10.1016/j.ophtha.2010.02.031 PMC2937272

[35] Bressler SB, CHANGES IN DIABETIC RETINOPATHY SEVERITY WHEN TREATING DIABETIC MACULAR EDEMA WITH RANIBIZUMAB. Oct.,, *Retina*, vol. 38, no. 10, pp. 1896–1904, 2018, 10.1097/IAE.0000000000002302 10.1097/IAE.0000000000002302 PMC6547828 30234859

[36] Lang GE, et al. Two-Year Safety and Efficacy of Ranibizumab 0.5 mg in Diabetic Macular Edema. Ophthalmology. Oct. 2013;120(10):2004–12. 10.1016/j.ophtha.2013.02.019. 10.1016/j.ophtha.2013.02.019 23725735

[37] Massin P et al. Nov., Safety and Efficacy of Ranibizumab in Diabetic Macular Edema (RESOLVE Study), *Diabetes Care*, vol. 33, no. 11, pp. 2399–2405, 2010, 10.2337/dc10-0493 10.2337/dc10-0493 PMC2963502 20980427

[38] Prünte C et al. Jun., Ranibizumab 0.5 mg treat-and-extend regimen for diabetic macular oedema: the RETAIN study, *Br. J. Ophthalmol.*, vol. 100, no. 6, pp. 787–795, 2016, 10.1136/bjophthalmol-2015-307249 10.1136/bjophthalmol-2015-307249 PMC4893084 26453639

[39] Joachim SC, et al. Protective effects on the retina after ranibizumab treatment in an ischemia model. PLoS ONE. Aug. 2017;12(8):e0182407. 10.1371/journal.pone.0182407. 10.1371/journal.pone.0182407 PMC5553852 28800629

[40] Holekamp NM et al. Mar., Archway Randomized Phase 3 Trial of the Port Delivery System with Ranibizumab for Neovascular Age-Related Macular Degeneration, *Ophthalmology*, vol. 129, no. 3, pp. 295–307, 2022, 10.1016/j.ophtha.2021.09.016 10.1016/j.ophtha.2021.09.016 34597713

[41] Campochiaro PA et al. Aug., The Port Delivery System with Ranibizumab for Neovascular Age-Related Macular Degeneration, *Ophthalmology*, vol. 126, no. 8, pp. 1141–1154, 2019, 10.1016/j.ophtha.2019.03.036 10.1016/j.ophtha.2019.03.036 30946888

[42] Chang Y-C et al. Mar., Optical Coherence Tomography Biomarkers in Predicting Treatment Outcomes of Diabetic Macular Edema after Ranibizumab Injections, *Medicina (B. Aires).*, vol. 59, no. 3, p. 629, 2023, 10.3390/medicina59030629 10.3390/medicina59030629 PMC10053215 36984630

[43] Hasan YTN, et al. Evaluation of marker-based optical coherence tomography findings in diabetic macular edema treated with intravitreal ranibizumab therapy. Rev Médica Clínica Las Condes. May 2023;34(3):187–94. 10.1016/j.rmclc.2023.04.001.

[44] Ghasemi Falavarjani K, Nguyen QD. Adverse events and complications associated with intravitreal injection of anti-VEGF agents: a review of literature, *Eye*, vol. 27, no. 7, pp. 787–794, Jul. 2013, 10.1038/eye.2013.107 10.1038/eye.2013.107 PMC3709385 23722722

[45] Lang GE, et al. The RELATION study: efficacy and safety of ranibizumab combined with laser photocoagulation treatment versus laser monotherapy in NPDR and PDR patients with diabetic macular oedema. Acta Ophthalmol. May 2018;96(3):e377–85. 10.1111/aos.13574. 10.1111/aos.13574 PMC5947710 29090846

[46] Zarbin MA, et al. Vascular safety of Ranibizumab in patients with Diabetic Macular Edema. JAMA Ophthalmol. May 2017;135(5):424. 10.1001/jamaophthalmol.2017.0455. 10.1001/jamaophthalmol.2017.0455 PMC5847109 28384675

[47] Chen C-H, et al. Intravitreal ranibizumab injection is associated with an increased risk of chronic kidney disease: a population-based study in Taiwan. Naunyn Schmiedebergs Arch Pharmacol. Dec. 2023. 10.1007/s00210-023-02910-x. 10.1007/s00210-023-02910-x PMC11166851 38153512

[48] Smolen JS, Goncalves J, Quinn M, Benedetti F, Lee JY. Era of biosimilars in rheumatology: reshaping the healthcare environment, *RMD Open*, vol. 5, no. 1, p. e000900, May 2019, 10.1136/rmdopen-2019-000900 10.1136/rmdopen-2019-000900 PMC6560670 31245050

[49] Sharma S, et al. Safety and efficacy of Razumab™ (world’s first biosimilar ranibizumab) in wet age-related macular degeneration: a post-marketing, prospective ASSET study. Int J Retin Vitr. Dec. 2021;7(1):24. 10.1186/s40942-021-00293-w. 10.1186/s40942-021-00293-w PMC7992797 33762008

[50] Holz FG, Oleksy P, Ricci F, Kaiser PK, Kiefer J, Schmitz-Valckenberg S. Efficacy and safety of Biosimilar FYB201 compared with Ranibizumab in Neovascular Age-Related Macular Degeneration. Ophthalmology. Jan. 2022;129(1):54–63. 10.1016/j.ophtha.2021.04.031. 10.1016/j.ophtha.2021.04.031 33957183

[51] Sharma A, Kumar N, Parachuri N. Biosimilar Ranibizumab (SB11) vs reference ranibizumab—diving deeper for Safety and Efficacy. JAMA Ophthalmol. Jun. 2021;139(6):677. 10.1001/jamaophthalmol.2021.1037. 10.1001/jamaophthalmol.2021.1037 33914017

[52] Cao Y. Positive and negative modulation of angiogenesis by VEGFR1 ligands. Sci Signal. Feb. 2009;2(59). 10.1126/scisignal.259re1. 10.1126/scisignal.259re1 19244214

[53] Papadopoulos N, et al. Binding and neutralization of vascular endothelial growth factor (VEGF) and related ligands by VEGF trap, ranibizumab and bevacizumab. Angiogenesis. Jun. 2012;15(2):171–85. 10.1007/s10456-011-9249-6. 10.1007/s10456-011-9249-6 PMC3338918 22302382

[54] Heier JS, et al. Intravitreal Aflibercept for Diabetic Macular Edema. Ophthalmology. Nov. 2016;123(11):2376–85. 10.1016/j.ophtha.2016.07.032. 10.1016/j.ophtha.2016.07.032 27651226

[55] Stahl A, et al. Systemic exposure to aflibercept after intravitreal injection in premature neonates with retinopathy of prematurity: results from the FIREFLEYE randomized phase 3 study. Eye Jan. 2024. 10.1038/s41433-023-02919-9. 10.1038/s41433-023-02919-9 PMC11126565 38200320

[56] Aflibercept. Bevacizumab, or Ranibizumab for Diabetic Macular Edema, *N. Engl. J. Med.*, vol. 372, no. 13, pp. 1193–1203, Mar. 2015, 10.1056/NEJMoa1414264 10.1056/NEJMoa1414264 PMC4422053 25692915

[57] Jampol LM, Glassman AR, Bressler NM, Wells JA, Ayala AR. Anti–vascular endothelial growth factor comparative effectiveness trial for Diabetic Macular Edema. JAMA Ophthalmol. Dec. 2016;134(12):1429. 10.1001/jamaophthalmol.2016.3698. 10.1001/jamaophthalmol.2016.3698 PMC5567802 27711918

[58] Do DV. Aflibercept 8 mg for Diabetic Macular Edema: 48-Week Results From the Phase 2/3 PHOTON Trial, *Invest. Ophthalmol. Vis. Sci.*, vol. 64, no. 8, p. 2814, Jun. 2023. 10.1016/j.ophtha.2025.10.028 41223900

[59] Ghorayeb G. Intravitreal Aflibercept 8 mg for Diabetic Macular Edema: Week 48 Efficacy Outcomes by Baseline Demographics in the Phase 2/3 PHOTON Trial, *Invest. Ophthalmol. Vis. Sci.*, vol. 64, no. 8, p. 2707, Jun. 2023.

[60] Chou Y-I, Chang H-Y, Lin M-Y, Tseng C-H, Wang T-J, Lin I-C. Risk analysis for patients with arterial thromboembolic events after intravitreal ranibizumab or aflibercept injections. Sci Rep. May 2023;13(1):7597. 10.1038/s41598-023-34128-5. 10.1038/s41598-023-34128-5 PMC10172364 37165045

[61] Kang EY-C, et al. The Association of Intravitreal Injections of different anti-vascular endothelial growth factor with systemic outcomes in Diabetic patients. J Pers Med. Mar. 2023;13(3):544. 10.3390/jpm13030544. 10.3390/jpm13030544 PMC10057023 36983725

[62] Feng HL, Abdelwahab S, Imam N, Astafurov K, Roth DB. Reduced Incidence of Intravitreal Injection–Related Endophthalmitis With Prefilled Syringes, *J. Vitreoretin. Dis.*, vol. 7, no. 4, pp. 305–309, Jul. 2023, 10.1177/24741264231159011 10.1177/24741264231159011 PMC10621703 37927312

[63] Kvopka M, Chan W, Baranage D, Sia D. Morganella morganii and Enterococcus faecalis endophthalmitis following intravitreal injection. BMC Ophthalmol. Nov. 2023;23(1):450. 10.1186/s12886-023-03198-4. 10.1186/s12886-023-03198-4 PMC10638747 37950172

[64] Ton N-S, Goncharov V, Zapata I, Adam MK. Endophthalmitis after Anti-VEGF Intravitreal injections with aqueous chlorhexidine versus povidone–iodine as ocular antiseptics. Ophthalmol Retin. Dec. 2023. 10.1016/j.oret.2023.12.004. 10.1016/j.oret.2023.12.004 38122867

[65] Russell MW et al. Nov., Effect of Prefilled vs Vial-Drawn Syringes on Sustained Increases in Intraocular Pressure in Patients Treated With Aflibercept, *J. Vitreoretin. Dis.*, vol. 7, no. 6, pp. 498–503, 2023, 10.1177/24741264231200735 10.1177/24741264231200735 PMC10649452 37974923

[66] Grimaldi G, et al. Intravitreal faricimab for neovascular age-related macular degeneration previously treated with traditional anti-VEGF compounds: a real-world prospective study. Graefe’s Arch Clin Exp Ophthalmol. Dec. 2023. 10.1007/s00417-023-06319-3. 10.1007/s00417-023-06319-3 38047930

[67] Ohara H, Harada Y, Hiyama T, Sadahide A, Minamoto A, Kiuchi Y. Faricimab for Diabetic Macular Edema in patients refractory to Ranibizumab or Aflibercept. Med (B Aires). Jun. 2023;59(6):1125. 10.3390/medicina59061125. 10.3390/medicina59061125 PMC10302733 37374329

[68] Rahman EZ, Singer MA. Brolucizumab as treatment of wet age-related maculopathy. Drugs Today. 2020;56(11):699. 10.1358/dot.2020.56.11.3199812. 10.1358/dot.2020.56.11.3199812 33332477

[69] Nguyen QD, et al. Brolucizumab: evolution through preclinical and clinical studies and the implications for the management of Neovascular Age-Related Macular Degeneration. Ophthalmology. Jul. 2020;127(7):963–76. 10.1016/j.ophtha.2019.12.031. 10.1016/j.ophtha.2019.12.031 32107066

[70] Holz FG, et al. Single-chain antibody fragment VEGF inhibitor RTH258 for Neovascular Age-Related Macular Degeneration. Ophthalmology. May 2016;123(5):1080–9. 10.1016/j.ophtha.2015.12.030. 10.1016/j.ophtha.2015.12.030 26906165

[71] Dugel PU et al. HAWK and HARRIER: Ninety-Six-Week Outcomes from the Phase 3 Trials of Brolucizumab for Neovascular Age-Related Macular Degeneration, *Ophthalmology*, vol. 128, no. 1, pp. 89–99, 2021, 10.1016/j.ophtha.2020.06.028 10.1016/j.ophtha.2020.06.028 32574761

[72] Wykoff CC et al. KESTREL and KITE Phase 3 Studies: 100-Week Results With Brolucizumab in Patients With Diabetic Macular Edema, *Am. J. Ophthalmol.*, vol. 260, pp. 70–83, Apr. 2024, 10.1016/j.ajo.2023.07.012 10.1016/j.ajo.2023.07.012 37460036

[73] Chauhan MZ, Rather PA, Samarah SM, Elhusseiny AM, Sallam AB. Current and Novel Therapeutic Approaches for Treatment of Diabetic Macular Edema, *Cells*, vol. 11, no. 12, p. 1950, Jun. 2022, 10.3390/cells11121950 10.3390/cells11121950 PMC9221813 35741079

[74] Witkin AJ et al. Jul., Occlusive Retinal Vasculitis Following Intravitreal Brolucizumab, *J. Vitreoretin. Dis.*, vol. 4, no. 4, pp. 269–279, 2020, 10.1177/2474126420930863 10.1177/2474126420930863 PMC7418897 32789284

[75] Hirano T, Toriyama Y, Takahashi Y, Hoshiyama K, Murata T. Retinal arterial occlusive vasculitis after multiple intravitreal brolucizumab injections for diabetic macular edema. Am J Ophthalmol Case Rep. Mar. 2023;29:101788. 10.1016/j.ajoc.2022.101788. 10.1016/j.ajoc.2022.101788 PMC9826871 36632338

[76] Chionh F, et al. VEGF-A, VEGFR1 and VEGFR2 single nucleotide polymorphisms and outcomes from the AGITG MAX trial of capecitabine, bevacizumab and mitomycin C in metastatic colorectal cancer. Sci Rep. Jan. 2022;12(1):1238. 10.1038/s41598-021-03952-y. 10.1038/s41598-021-03952-y PMC8786898 35075138

[77] Gomez-Lumbreras A, Ghule P, Panchal R, Giannouchos T, Lockhart CM, Brixner D. Real-world evidence in the use of Bevacizumab in age-related macular degeneration (ArMD): a scoping review, *Int. Ophthalmol.*, vol. 43, no. 12, pp. 4527–4539, Aug. 2023, 10.1007/s10792-023-02853-5 10.1007/s10792-023-02853-5 37606820

[78] Kaiser PK, Schmitz-Valckenberg MS, Holz FG, ANTI-VASCULAR ENDOTHELIAL GROWTH FACTOR BIOSIMILARS IN OPHTHALMOLOGY. Dec., *Retina*, vol. 42, no. 12, pp. 2243–2250, 2022, 10.1097/IAE.0000000000003626 10.1097/IAE.0000000000003626 PMC9665947 36394884

[79] Chakravarthy U, et al. Alternative treatments to inhibit VEGF in age-related choroidal neovascularisation: 2-year findings of the IVAN randomised controlled trial. Lancet. Oct. 2013;382(9900):1258–67. 10.1016/S0140-6736(13)61501-9. 10.1016/S0140-6736(13)61501-9 23870813

[80] Stein JD, Newman-Casey PA, Kim DD, Nwanyanwu KH, Johnson MW, Hutton DW. Cost-Effectiveness of Various Interventions for Newly Diagnosed Diabetic Macular Edema, *Ophthalmology*, vol. 120, no. 9, pp. 1835–1842, Sep. 2013, 10.1016/j.ophtha.2013.02.002 10.1016/j.ophtha.2013.02.002 PMC3737388 23642372

[81] Sharma A, Kumar N, Parachuri N, Loewenstein A, Bandello F, Kuppermann BD. On label bevacizumab for retina: where it stands. Eye. May 2022;36(5):916–7. 10.1038/s41433-021-01909-z. 10.1038/s41433-021-01909-z PMC9046423 35046548

[82] Therapeutics O. Outlook Therapeutics Presents NORSE TWO Phase 3 Pivotal Safety and Efficacy Data for ONS-5010 / LYTENAVA ^TM^ (bevacizumab-vikg) at the Retina Subspecialty Day, American Academy of Ophthalmology (AAO) 2021 Annual Conference, 2022.

[83] Jhaveri CD et al. Aug., Aflibercept Monotherapy or Bevacizumab First for Diabetic Macular Edema, *N. Engl. J. Med.*, vol. 387, no. 8, pp. 692–703, 2022, 10.1056/NEJMoa2204225 10.1056/NEJMoa2204225 PMC9714135 35833805

[84] Fekri S, Soheilian M, Roozdar S, Abtahi S-H, Nouri H. The effect of vitamin D supplementation on the outcome of treatment with bevacizumab in diabetic macular edema: a randomized clinical trial. Int Ophthalmol. May 2022;42(11):3345–56. 10.1007/s10792-022-02333-2. 10.1007/s10792-022-02333-2 PMC9093557 35543853

[85] The royal college of ophthalmologists. nice final draft guidance recommending new drug faricimab to help treat two leading causes of sight loss and visual impairment 09 jun 2022 the royal college of ophthalmologists, 2022. https://www.rcophth.ac.uk/news-views/nice-final-draft-guidance-recommending-new-drug-faricimab-to-help-treat-two-leading-causes-of-sight-loss-and-visual-impairment/#:text=Other useful links-,NICE final draft guidance recommending new drug faricimab to h.

[86] AAO, Faricimab. Latest Research Results, 2023, [Online]. Available: https://www.aao.org/eyenet/academy-live/detail/faricimab-latest-research-results

[87] Maisonpierre PC et al. Jul., Angiopoietin-2, a Natural Antagonist for Tie2 That Disrupts in vivo Angiogenesis, *Science (80-.).*, vol. 277, no. 5322, pp. 55–60, 1997, 10.1126/science.277.5322.55 10.1126/science.277.5322.55 9204896

[88] Fiedler U et al. Feb., Angiopoietin-2 sensitizes endothelial cells to TNF-α and has a crucial role in the induction of inflammation, *Nat. Med.*, vol. 12, no. 2, pp. 235–239, 2006, 10.1038/nm1351 10.1038/nm1351 16462802

[89] Klein C et al. Feb., Engineering therapeutic bispecific antibodies using CrossMab technology, *Methods*, vol. 154, pp. 21–31, 2019, 10.1016/j.ymeth.2018.11.008 10.1016/j.ymeth.2018.11.008 30453028

[90] Collazos-Alemán JD, Gnecco-González S, Jaramillo-Zarama B, Jiménez-Mora MA, Mendivil CO. The Role of Angiopoietins in Neovascular Diabetes-Related Retinal Diseases, *Diabetes Ther.*, vol. 13, no. 11–12, pp. 1811–1821, Dec. 2022, 10.1007/s13300-022-01326-9 10.1007/s13300-022-01326-9 PMC9663771 36331711

[91] Rush RB. One Year Results of Faricimab for Aflibercept-Resistant Diabetic Macular Edema, *Clin. Ophthalmol.*, vol. Volume 17, pp. 2397–2403, Aug. 2023, 10.2147/OPTH.S424314 10.2147/OPTH.S424314 PMC10440101 37605765

[92] Liberski S, Wichrowska M, Kocięcki J. Aflibercept versus Faricimab in the treatment of Neovascular Age-Related Macular Degeneration and Diabetic Macular Edema: a review. Int J Mol Sci. Aug. 2022;23(16):9424. 10.3390/ijms23169424. 10.3390/ijms23169424 PMC9409486 36012690

[93] Li G, Zhu N, Ji A. Comparative efficacy and safety of Faricimab and other anti-VEGF therapy for age-related macular degeneration and diabetic macular edema: A systematic review and meta-analysis of randomized clinical trials, *Medicine (Baltimore).*, vol. 102, no. 50, p. e36370, Dec. 2023, 10.1097/MD.0000000000036370 10.1097/MD.0000000000036370 PMC10727610 38115358

[94] Takamura Y, Yamada Y, Morioka M, Gozawa M, Matsumura T, Inatani M. Turnover of Microaneurysms after Intravitreal injections of Faricimab for Diabetic Macular Edema. Investig Opthalmology Vis Sci. 2023;64. 10.1167/iovs.64.13.31. 13, p. 31, Oct. 10.1167/iovs.64.13.31 PMC10593137 37856112

[95] Lu X, Sun X. Profile of conbercept in the treatment of neovascular age-related macular degeneration, *Drug Des. Devel. Ther.*, vol. 9, pp. 2311–2320, Apr. 2015, 10.2147/DDDT.S67536 10.2147/DDDT.S67536 PMC4410828 25960634

[96] Liu K et al. Oct., Intravitreal conbercept for diabetic macular oedema: 2-year results from a randomised controlled trial and open-label extension study, *Br. J. Ophthalmol.*, vol. 106, no. 10, pp. 1436–1443, 2022, 10.1136/bjophthalmol-2020-318690 10.1136/bjophthalmol-2020-318690 PMC9510409 34001667

[97] Ba T, et al. Evaluation of the efficacy of Conbercept in the treatment of diabetic macular edema based on OCTA. Med (Baltim). Aug. 2020;99:e21992. 10.1097/MD.0000000000021992. no. 35. 10.1097/MD.0000000000021992 PMC7458221 32871950

[98] Liu W, Li Y, Cao R, Bai Z, Liu W. A systematic review and meta-analysis to compare the efficacy of conbercept with ranibizumab in patients with macular edema secondary to retinal vein occlusion. Med (Baltim). May 2020;99(21):e20222. 10.1097/MD.0000000000020222. 10.1097/MD.0000000000020222 PMC7249991 32481293

[99] Wang H, et al. One-Year Effectiveness Study of Intravitreously Administered Conbercept^®^ Monotherapy in Diabetic Macular Degeneration: A Systematic Review and Meta-Analysis. Diabetes Ther. May 2020;11(5):1103–17. 10.1007/s13300-020-00806-0. 10.1007/s13300-020-00806-0 PMC7192996 32236812

[100] Wang X, Yu C, Yang J, Liu Y, Xu Y, Li W. Comparison of efficacy and safety between Conbercept and Ranibizumab in Neovascular Age-Related Macular Degeneration: a Meta-analysis of Randomized controlled trials. Ophthalmic Res. 2022;65(2):140–51. 10.1159/000519815. 34583363 10.1159/000519815

[101] Xing P, Meng B, Hu X, Qu W, Wang S. Switching to Conbercept in Diabetic Macular Edema After Unsatisfactory Response to Previous Intravitreal Injection of Ranibizumab, *Clin. Ophthalmol.*, vol. Volume 17, pp. 3491–3497, Nov. 2023, 10.2147/OPTH.S431145 10.2147/OPTH.S431145 PMC10661898 38026602

[102] Zhu Z, et al. Clinical effect of conbercept on improving diabetic macular ischemia by OCT angiography. BMC Ophthalmol. Dec. 2020;20(1):382. 10.1186/s12886-020-01648-x. 10.1186/s12886-020-01648-x PMC7519504 32977791

[103] Gonzalez-Cortes JH, et al. Current treatments and innovations in Diabetic Retinopathy and Diabetic Macular Edema. Pharmaceutics. Dec. 2022;15(1). 10.3390/pharmaceutics15010122. 10.3390/pharmaceutics15010122 PMC9866607 36678750

[104] Exploring neovascular age. -related macular degeneration and diabetic macular edema and advances in treatment. Am J Manag Care. Mar. 2022;28:S35–43. 10.37765/ajmc.2022.88853. 10.37765/ajmc.2022.88853 35275609

[105] Aceves-Franco LA, Sanchez-Aguilar OE, Barragan-Arias AR, Ponce-Gallegos MA, Navarro-Partida J, Santos A. The Evolution of Triamcinolone Acetonide Therapeutic Use in Retinal Diseases: From Off-Label Intravitreal Injection to Advanced Nano-Drug Delivery Systems, *Biomedicines*, vol. 11, no. 7, p. 1901, Jul. 2023, 10.3390/biomedicines11071901 10.3390/biomedicines11071901 PMC10377205 37509540

[106] Kodjikian L et al. Jul., Fluocinolone acetonide implant in diabetic macular edema: International experts’ panel consensus guidelines and treatment algorithm., *Eur. J. Ophthalmol.*, vol. 32, no. 4, pp. 1890–1899, 2022, 10.1177/11206721221080288 10.1177/11206721221080288 35139688

[107] Kato F, Nozaki M, Kato A, Yasukawa T. Retinal microvascular changes after Intravitreal Triamcinolone Acetonide in Diabetic Macular Edema. J Clin Med. May 2023;12(10):3475. 10.3390/jcm12103475. 10.3390/jcm12103475 PMC10219239 37240582

[108] Nawar AE. Effectiveness of Suprachoroidal Injection of Triamcinolone Acetonide in Resistant Diabetic Macular Edema Using a Modified Microneedle, *Clin. Ophthalmol.*, vol. Volume 16, pp. 3821–3831, Nov. 2022, 10.2147/OPTH.S391319 10.2147/OPTH.S391319 PMC9698330 36438589

[109] Carreira AR, et al. Safety of intravitreal triamcinolone and its impact on optic nerve morphology in patients treated for diabetic macular edema. Eur J Ophthalmol. May 2022;32(3):1596–601. 10.1177/11206721211028744. 10.1177/11206721211028744 34176301

[110] Dang Y, et al. Comparison of dexamethasone intravitreal implant and intravitreal triamcinolone acetonide for the treatment of pseudophakic cystoid macular edema in diabetic patients. Drug Des Devel Ther. 2014;8:1441–9. 10.2147/DDDT.S66611. 25258512 10.2147/DDDT.S66611 PMC4174031

[111] Bux AV, Fortunato F, Barone A, Russo V, Delle Noci N, Iaculli C. Early treatment with dexamethasone intravitreal implants in diabetic macular edema: Naïve versus refractory patients. Eur J Ophthalmol. May 2022;32(3):1619–26. 10.1177/11206721211024804. 10.1177/11206721211024804 34120496

[112] Kuley B, et al. Treatment of eyes with Diabetic Macular Edema that had a suboptimal response to Antivascular endothelial growth factor therapy: 2-mg Intravitreal Triamcinolone Acetonide vs 0.7-mg Dexamethasone Implant. J Vitreoretin Dis. 2020;4(5):372–6. 10.1177/2474126420917268. 37008292 10.1177/2474126420917268 PMC9979017

[113] Ciulla TA, Harris A, McIntyre N, Jonescu-Cuypers C. Treatment of diabetic macular edema with sustained-release glucocorticoids: intravitreal triamcinolone acetonide, dexamethasone implant, and fluocinolone acetonide implant. Expert Opin Pharmacother. May 2014;15(7):953–9. 10.1517/14656566.2014.896899. 10.1517/14656566.2014.896899 24661081

[114] Kolomeyer AM, Eichenbaum DA, Kiernan DF, Suñer IJ, Hariprasad SM. The 0.19-mg Fluocinolone Acetonide Implant for the Treatment of Diabetic Macular Edema: An Expert Consensus, *Ophthalmic Surgery, Lasers Imaging Retin.*, vol. 54, no. 3, pp. 166–173, Mar. 2023, 10.3928/23258160-20230215-01 10.3928/23258160-20230215-01 36944067

[115] Fleissig E, Sigford DK. Effect of Extended Release Steroid implants on the Contralateral Eye. BMC Ophthalmol. Dec. 2022;22(1):131. 10.1186/s12886-022-02357-3. 10.1186/s12886-022-02357-3 PMC8939114 35317754

[116] Kume A, Kashiwagi K. Systemic and ocular diseases associated with the development of diabetic macular edema among Japanese patients with diabetes mellitus. BMC Ophthalmol. Dec. 2020;20(1):309. 10.1186/s12886-020-01578-8. 10.1186/s12886-020-01578-8 PMC7392833 32727408

[117] Acan D, et al. The prevalence and systemic risk factors of diabetic macular edema: a cross-sectional study from Turkey. BMC Ophthalmol. Dec. 2018;18(1):91. 10.1186/s12886-018-0753-y. 10.1186/s12886-018-0753-y PMC5897948 29649995

[118] Xie J, et al. Association of Diabetic Macular Edema and proliferative Diabetic Retinopathy with Cardiovascular Disease. JAMA Ophthalmol. Jun. 2017;135(6):586. 10.1001/jamaophthalmol.2017.0988. 10.1001/jamaophthalmol.2017.0988 PMC5593137 28472362

[119] Dicembrini I, et al. Microvascular effects of glucagon-like peptide-1 receptor agonists in type 2 diabetes: a meta-analysis of randomized controlled trials. Acta Diabetol. Oct. 2017;54(10):933–41. 10.1007/s00592-017-1031-9. 10.1007/s00592-017-1031-9 28748377

[120] Fong DS, Contreras R. Glitazone Use Associated with Diabetic Macular Edema. Am J Ophthalmol. 2009;147(4):583–6. 10.1016/j.ajo.2008.10.016. .e1, Apr. 10.1016/j.ajo.2008.10.016 19181303

[121] Diep TM, Tsui I. Risk factors associated with diabetic macular edema. Diabetes Res Clin Pract. Jun. 2013;100(3):298–305. 10.1016/j.diabres.2013.01.011. 10.1016/j.diabres.2013.01.011 23380134

[122] An J. Insulin Use and Risk of Diabetic Macular Edema in Diabetes Mellitus: a systemic review and Meta-analysis of Observational studies. Med Sci Monit. 2015;21:929–36. 10.12659/MSM.892056. 25816765 10.12659/MSM.892056 PMC4384512

[123] RYAN EH, May., DIABETIC MACULAR EDEMA ASSOCIATED WITH GLITAZONE USE., *Retina*, vol. 26, no. 5, pp. 562–570, 2006, 10.1097/00006982-200605000-00011 10.1097/00006982-200605000-00011 16770264

[124] Haq S et al. Jan., Characterization of the Systemic Findings of Patients Undergoing Initiation of Anti-Vascular Endothelial Growth Factor Therapy for Diabetic Macular Edema in Routine Clinical Practice, *Ophthalmic Surgery, Lasers Imaging Retin.*, vol. 50, no. 1, pp. 16–24, 2019, 10.3928/23258160-20181212-03 10.3928/23258160-20181212-03 30640391

[125] Su Y et al. Sep., Risk of diabetic macular oedema with sodium-glucose cotransporter‐2 inhibitors in type 2 diabetes patients: A multi‐institutional cohort study in < scp > Taiwan, *Diabetes, Obes. Metab.*, vol. 23, no. 9, pp. 2067–2076, 2021, 10.1111/dom.14445 10.1111/dom.14445 34047442

[126] Rahimy E, Baker K, Thompson D, Saroj N. Impact of Systemic Dipeptidyl Peptidase-4 Inhibitor Use in Diabetic Macular Edema, *Ophthalmic Surgery, Lasers Imaging Retin.*, vol. 51, no. 4, pp. 226–234, Apr. 2020, 10.3928/23258160-20200326-04 10.3928/23258160-20200326-04 32348539

[127] Chung Y-R, et al. Association of statin use and hypertriglyceridemia with diabetic macular edema in patients with type 2 diabetes and diabetic retinopathy. Cardiovasc Diabetol. Dec. 2017;16(1). 10.1186/s12933-016-0486-2. 10.1186/s12933-016-0486-2 PMC5219811 28061854

[128] Mozetic V, Pacheco RL, de Latorraca C, Riera R. Statins and/or fibrates for diabetic retinopathy: a systematic review and meta-analysis. Diabetol Metab Syndr. Dec. 2019;11(1):92. 10.1186/s13098-019-0488-9. 10.1186/s13098-019-0488-9 PMC6839185 31719846

[129] Meer E, Bavinger JC, Yu Y, VanderBeek BL. Association of Fenofibrate Use and the risk of progression to Vision-threatening Diabetic Retinopathy. JAMA Ophthalmol. May 2022;140(5):529. 10.1001/jamaophthalmol.2022.0633. 10.1001/jamaophthalmol.2022.0633 PMC8990357 35389455

[130] Pascual-Camps I, et al. Update on intravitreal anti-tumor necrosis factor alpha therapies for ocular disorders. J Ophthalmic Inflamm Infect. Dec. 2014;4(1). 10.1186/s12348-014-0026-8. 10.1186/s12348-014-0026-8 PMC4372686 25825604

[131] GIGANTI M, BEER PM, LEMANSKI N, HARTMAN C, J. SCHARTMAN, and, FALK N, ADVERSE EVENTS AFTER INTRAVITREAL INFLIXIMAB (REMICADE). Retina. Jan. 2010;30(1):71–80. 10.1097/IAE.0b013e3181bcef3b. 10.1097/IAE.0b013e3181bcef3b 19996827

[132] Sfikakis PP et al. Jul., Infliximab for Diabetic Macular Edema Refractory to Laser Photocoagulation, *Diabetes Care*, vol. 33, no. 7, pp. 1523–1528, 2010, 10.2337/dc09-2372 10.2337/dc09-2372 PMC2890353 20413522

[133] Sharifipour F, et al. Systemic oxygen therapy versus oral enalapril for treatment of diabetic macular ischemia: a randomized controlled trial. Int Ophthalmol. Apr. 2016;36(2):225–35. 10.1007/s10792-015-0123-1. 10.1007/s10792-015-0123-1 26292645

[134] Maalej A, Khallouli A, Choura R, Ben Sassi R, Rannen R, Gharsallah H. The effects of hyperbaric oxygen therapy on diabetic retinopathy: A preliminary study, *J. Fr. Ophtalmol.*, vol. 43, no. 2, pp. 133–138, Feb. 2020, 10.1016/j.jfo.2019.07.005 10.1016/j.jfo.2019.07.005 31831276

[135] Sellman A, Katzman P, Andreasson S, Löndahl M. Long-term effects of hyperbaric oxygen therapy on visual acuity and retinopathy, *Undersea Hyperb. Med.*, pp. 423–430, Mar. 2020, 10.22462/03.07.2020.3 32931668

[136] Munnerlyn CR. Lasers in opthalmology: Past, present and future, *J. Mod. Opt.*, vol. 50–15, no. 17, pp. 2351–2360, Oct. 2003, 10.1080/09500340308233566

[137] Nozaki M, Ando R, Kimura T, Kato F, Yasukawa T. The Role of Laser Photocoagulation in Treating Diabetic Macular Edema in the Era of Intravitreal Drug Administration: A Descriptive Review, *Medicina (B. Aires).*, vol. 59, no. 7, p. 1319, Jul. 2023, 10.3390/medicina59071319 10.3390/medicina59071319 PMC10385537 37512130

[138] Patz A, Smith RE, The ETDRS and, Diabetes. 2000, *Ophthalmology*, vol. 98, no. 5, pp. 739–740, May 1991, 10.1016/S0161-6420(13)38007-5 10.1016/s0161-6420(13)38007-5 2062509

[139] Everett LA, Paulus YM. Laser therapy in the treatment of Diabetic Retinopathy and Diabetic Macular Edema. Curr Diab Rep. Sep. 2021;21:35. 10.1007/s11892-021-01403-6. 10.1007/s11892-021-01403-6 PMC8420141 34487257

[140] Schatz H. Progressive Enlargement of Laser Scars Following Grid Laser Photocoagulation for Diffuse Diabetic Macular Edema. Arch Ophthalmol. Nov. 1991;109(11):1549. 10.1001/archopht.1991.01080110085041. 10.1001/archopht.1991.01080110085041 1755735

[141] Roider J. Laser Treatment of Retinal diseases by Subthreshold Laser effects. Semin Ophthalmol. Jan. 1999;14(1):19–26. 10.3109/08820539909056059. 10.3109/08820539909056059 10790572

[142] Romero-Aroca P, Reyes-Torres J, Baget-Bernaldiz M, Blasco-Sune C. Laser Treatment for Diabetic Macular Edema in the 21st Century. Curr Diabetes Rev. May 2014;10(2):100–12. 10.2174/1573399810666140402123026. 10.2174/1573399810666140402123026 PMC4051253 24852439

[143] Paulus YM, Kaur K, Egbert PR, Blumenkranz MS, Moshfeghi DM. Human histopathology of PASCAL laser burns, *Eye*, vol. 27, no. 8, pp. 995–996, Aug. 2013, 10.1038/eye.2013.100 10.1038/eye.2013.100 PMC3740304 23722723

[144] Muqit MMK et al. Nov., Pain responses of Pascal 20 ms multi-spot and 100 ms single-spot panretinal photocoagulation: Manchester Pascal Study, MAPASS report 2, *Br. J. Ophthalmol.*, vol. 94, no. 11, pp. 1493–1498, 2010, 10.1136/bjo.2009.176677 10.1136/bjo.2009.176677 20558423

[145] Muqit MMK, et al. Barely visible 10-Millisecond Pascal Laser Photocoagulation for Diabetic Macular Edema: observations of clinical effect and burn localization. Am J Ophthalmol. Jun. 2010;149:979–e9862. 10.1016/j.ajo.2010.01.032. 10.1016/j.ajo.2010.01.032 20510687

[146] Muqit MMK et al. Mar., Study of clinical applications and safety for Pascal ^®^ laser photocoagulation in retinal vascular disorders, *Acta Ophthalmol.*, vol. 90, no. 2, pp. 155–161, 2012, 10.1111/j.1755-3768.2009.01854.x 10.1111/j.1755-3768.2009.01854.x 20163363

[147] Lavinsky D, Cardillo JA, Melo LAS, Dare A, Farah ME, Belfort R. Randomized Clinical Trial evaluating mETDRS versus normal or high-density Micropulse Photocoagulation for Diabetic Macular Edema. Investig Opthalmology Vis Sci. Jun. 2011;52(7):4314. 10.1167/iovs.10-6828. 10.1167/iovs.10-6828 21345996

[148] Frizziero L, et al. Diabetic Macular Edema Treated with 577-nm Subthreshold Micropulse laser: a Real-Life, long-term study. J Pers Med. May 2021;11(5):405. 10.3390/jpm11050405. 10.3390/jpm11050405 PMC8152245 34067994

[149] Vujosevic S, Bottega E, Casciano M, Pilotto E, Convento E, Midena E, MICROPERIMETRY AND FUNDUS AUTOFLUORESCENCE IN DIABETIC MACULAR EDEMA. Jun.,, *Retina*, vol. 30, no. 6, pp. 908–916, 2010, 10.1097/IAE.0b013e3181c96986 10.1097/IAE.0b013e3181c96986 20168272

[150] Cho HY, Kang SW, Kim YT, Chung SE, Lee SW. A three-year follow-up of Intravitreal Triamcinolone Acetonide Injection and Macular laser photocoagulation for diffuse Diabetic Macular Edema. Korean J Ophthalmol. 2012;26(5):362. 10.3341/kjo.2012.26.5.362. 23060723 10.3341/kjo.2012.26.5.362 PMC3464320

[151] Dourandeesh M, Moeini M, Shaye ZA, Shoeibi N, Hosseini SM, Banaee T. Subfoveal choroidal thickness following pars plana vitrectomy in tractional diabetic macular edema. Eur J Ophthalmol. May 2023;33(3):1405–11. 10.1177/11206721221144137. 10.1177/11206721221144137 36476066

[152] Ranno S, et al. Role of vitrectomy in Nontractional Refractory Diabetic Macular Edema. J Clin Med. Mar. 2023;12(6):2297. 10.3390/jcm12062297. 10.3390/jcm12062297 PMC10056256 36983298

[153] Iuliano L, Corbelli E, Bandello F, Codenotti M. Protective effect of vitrectomy on the course of diabetic retinopathy: a case report. Eur J Ophthalmol. Jan. 2022;32(1):NP177–80. 10.1177/1120672120968739. 10.1177/1120672120968739 33148051

[154] Khattab AAA, Ahmed MM, Hammed AH. Pars plana vitrectomy for tractional diabetic macular edema with or without internal limiting membrane peeling, *Med. hypothesis Discov. Innov. Ophthalmol.*, vol. 11, no. 3, pp. 110–118, Dec. 2022, 10.51329/mehdiophthal1454 10.51329/mehdiophthal1454 PMC10445315 37641643

[155] Guo H et al. Oct., Microinvasive pars plana vitrectomy combined with internal limiting membrane peeling versus anti-VEGF intravitreal injection for treatment-naïve diabetic macular edema (VVV-DME study): study protocol for a randomized controlled trial, *Trials*, vol. 24, no. 1, p. 685, 2023, 10.1186/s13063-023-07735-w 10.1186/s13063-023-07735-w PMC10594908 37875997

[156] Yan Y, THAN VITRECTOMY ALONE IN THE TREATMENT OF REFRACTORY DIABETIC MACULAR EDEMA. COMBINATION OF VITRECTOMY AND INTENTIONAL MACULAR DETACHMENT IS ASSOCIATED WITH A FASTER EDEMATOUS REGRESSION, *Retina*, vol. 42, no. 10, pp. 1859–1866, 2022, 10.1097/IAE.0000000000003536 10.1097/IAE.0000000000003536 36129263

[157] Franzolin E, Gusson E, Panozzo G. The effect of pars plana vitrectomy with internal limiting membrane peeling on the durability of the intravitreal dexamethasone implant in the treatment of diabetic macular edema, *Am. J. Ophthalmol. Case Reports*, vol. 26, p. 101401, Jun. 2022, 10.1016/j.ajoc.2022.101401 10.1016/j.ajoc.2022.101401 PMC8881408 35243151

[158] Chen J, Wang H, Qiu W. Intravitreal anti-vascular endothelial growth factor, laser photocoagulation, or combined therapy for diabetic macular edema: A systematic review and network meta-analysis, *Front. Endocrinol. (Lausanne).*, vol. 14, Feb. 2023, 10.3389/fendo.2023.1096105 10.3389/fendo.2023.1096105 PMC9933865 36817588

[159] Kuroiwa DAK, Malerbi FK, Regatieri CVS. New insights in Resistant Diabetic Macular Edema. Ophthalmologica. 2021;244(6):485–94. 10.1159/000516614. 34023834 10.1159/000516614

[160] Zhang L, Chen X. Efficacy and safety of triamcinolone acetonide injection combined with laser photocoagulation in the treatment of diabetic macular edema: a systematic review and meta-analysis, *Ann. Palliat. Med.*, vol. 10, no. 12, pp. 12467–12477, Dec. 2021, 10.21037/apm-21-3274 10.21037/apm-21-3274 35016410

[161] Liang J, Xiong T, Chen H, Tao R, Cao L. Effects of Ranibizumab combined with laser photocoagulation on macular volume and best corrected visual acuity in patients with macular edema secondary to ischemic retinal vein occlusion. Pakistan J Med Sci. Jan. 2023;39(2). 10.12669/pjms.39.2.7040. 10.12669/pjms.39.2.7040 PMC10025720 36950445

[162] Sadhukhan K. Role of combined therapy of Intravitreal Ranibizumab and Dexamethasone in Refractory Diabetic Macular Edema: a retrospective study. Maedica - J Clin Med. Dec. 2021;16(4). 10.26574/maedica.2021.16.4.615. 10.26574/maedica.2021.16.4.615 PMC8897799 35261663

[163] Jatoi AU, Shaikh NA, Narsan AK, Jatoi A. Intravitreal Bevacizumab alone Versus Intravitreal Bevacizumab in Combination with Focal Macular Photocoagulation in Diabetic Macular Oedema. J Ayub Med Coll Abbottabad. 2022;34(1):160–3. 10.55519/JAMC-01-9721. 35466645 10.55519/JAMC-01-9721

[164] Liu J, Li R, Han K, Yu X, Tang Y, Zhao H. Intravitreal injection of Conbercept combined with micropulse laser therapy enhances clinical efficacy in patients with diabetic macular edema., *Am. J. Transl. Res.*, vol. 15, no. 1, pp. 531–538, 2023, [Online]. Available: http://www.ncbi.nlm.nih.gov/pubmed/36777842 PMC9908468 36777842

[165] Qiao G. Sub-threshold micro-pulse diode laser treatment in diabetic macular edema: a Meta-analysis of randomized controlled trials. Int J Ophthalmol. Jul. 2016. 10.18240/ijo.2016.07.15. 10.18240/ijo.2016.07.15 PMC4951658 27500112

[166] Zhan H-Q, Zhou J-L, Zhang J, Wu D, Gu C-Y. Conbercept combined with laser photocoagulation in the treatment of diabetic macular edema and its influence on intraocular cytokines, *World J. Diabetes*, vol. 14, no. 8, pp. 1271–1279, Aug. 2023, 10.4239/wjd.v14.i8.1271 10.4239/wjd.v14.i8.1271 PMC10473943 37664482

[167] Ciulla TA, Bracha P, Pollack J, Williams DF. Real-world Outcomes of Anti–Vascular Endothelial Growth Factor Therapy in Diabetic Macular Edema in the United States, *Ophthalmol. Retin.*, vol. 2, no. 12, pp. 1179–1187, Dec. 2018, 10.1016/j.oret.2018.06.004 10.1016/j.oret.2018.06.004 31047187

[168] Bathini M, Raghushaker CR, Mahato KK. The Molecular mechanisms of Action of Photobiomodulation against neurodegenerative diseases: a systematic review. Cell Mol Neurobiol. May 2022;42(4):955–71. 10.1007/s10571-020-01016-9. 10.1007/s10571-020-01016-9 PMC8942959 33301129

[169] Johnstone D, Mitrofanis J, Stone J. Targeting the body to protect the brain: inducing neuroprotection with remotely-applied near infrared light. Neural Regen Res. 2015;10(3):349. 10.4103/1673-5374.153673. 25878572 10.4103/1673-5374.153673 PMC4396086

[170] Shen W, et al. Preclinical and clinical studies of photobiomodulation therapy for macular oedema. Diabetologia. Sep. 2020;63(9):1900–15. 10.1007/s00125-020-05189-2. 10.1007/s00125-020-05189-2 32661752

[171] Eells JT, Gopalakrishnan S, Valter K. Near-Infrared Photobiomodulation in Retinal Injury and Disease, 2016, pp. 437–41. 10.1007/978-3-319-17121-0_58 10.1007/978-3-319-17121-0_58 26427443

[172] Eells JT et al. Jun., 670 nm Photobiomodulation as a Therapy for Diabetic Macular Edema: A Pilot Study, *Invest. Ophthalmol. Vis. Sci.*, vol. 58, no. 8, p. 932, 2017.

[173] Kim JE, et al. A Randomized Trial of Photobiomodulation Therapy for Center-Involved Diabetic Macular Edema with Good Visual Acuity (Protocol AE). Ophthalmol Retin. Apr. 2022;6(4):298–307. 10.1016/j.oret.2021.10.003. 10.1016/j.oret.2021.10.003 PMC9011341 34628066

[174] Chandrasekaran PR, Madanagopalan VG. KSI-301: antibody biopolymer conjugate in retinal disorders. Ther Adv Ophthalmol. Jan. 2021;13:251584142110277. 10.1177/25158414211027708. 10.1177/25158414211027708 PMC8278447 34291186

[175] hong liang, et al. KSI-301: an anti-VEGF antibody biopolymer conjugate with extended half-life for treatment of neovascular retinal diseases. Invest Ophthalmol Vis Sci. Jul. 2018;59(9):211.

[176] Patel SS, et al. Phase 1 first-in-human study of KSI-301: a novel anti-VEGF antibody biopolymer conjugate with extended durability. Invest Ophthalmol Vis Sci. Jul. 2019;60(9):3670.

[177] Kodiak Sciences. Announces Topline results from its phase 3 studies of Tarcocimab Tedromer in Neovascular Age-Related Macular Degeneration and Diabetic Macular Edema and provides update on Tarcocimab Development Program; July 24, 2023. Accessed November 24.

[178] Cimberle M. Positive outcomes seen with combined OPT-302, aflibercept for DME in phase 2a trial. Healio News. November 17, 2020. Accessed September 24, 2023., 2020.

[179] Boyer D. Switching to combination OPT-302 with aflibercept from prior anti-VEGF-A monotherapy in eyes with persistent diabetic macula edema (DME). Presented at: American Society of Retina Specalists Virtual Meeting; July 25, 2020, 2020.

[180] Sozinibercept by Opthea for Diabetic Macular Edema. Likelihood of Approval. https://www.pharmaceutical-technology.com/data-insights/sozinibercept-opthea-diabetic-macular-edema-likelihood-of-approval-2, 2023.

[181] Kapur M, Nirula S, Naik MP. Future of anti-VEGF: biosimilars and biobetters, *Int. J. Retin. Vitr.*, vol. 8, no. 1, p. 2, Dec. 2022, 10.1186/s40942-021-00343-3 10.1186/s40942-021-00343-3 PMC8725244 34983660

[182] Innovent Presents Clinical Data of Two Ophthalmic Bispecific Antibodies IBI302 (anti-VEGF/complement). and IBI324 (anti-VEGF-A/Ang-2) at American Academy of Ophthalmology (AAO) Annual Meeting 2023, 2023. https://www.prnewswire.com/news-releases/innovent-presents-clinical-data-of-two-ophthalmic-bispecific-antibodies-ibi302-anti-vegfcomplement-and-ibi324-anti-vegf-aang-2-at-american-academy-of-ophthalmology-aao-annual-meeting-2023-301977847.html

[183] Phase 1 Study of IBI324. (Anti-VEGF-A/Ang-2 Bispecific Antibody) for Diabetic Macular Edema (DME). Abstract No.: PO524, 2023.

[184] Stefansson E et al. Feb., Topical treatment of diabetic macular edema using dexamethasone ophthalmic suspension: A randomized, double-masked, vehicle‐controlled study, *Acta Ophthalmol.*, vol. 101, no. 1, pp. 22–33, 2023, 10.1111/aos.15215 10.1111/aos.15215 35848336

[185] Oculis. Oculis announces positive top line results from DIAMOND Stage 1 Phase 3 Trial in diabetic macular edema with OCS-01 eye drops. Oculis. May 22. 2023. Accessed September 24, 2023., 2023, [Online]. Available: https://investors.oculis.com/news-releases/news-release-details/oculis-announces-positive-top-line-results-diamond-stage-1-phase/

[186] Emanuelli A et al. Jun., Port delivery system with ranibizumab in the treatment of diabetic retinopathy without center-involved diabetic macular edema: primary analysis results of the phase 3 Pavilion trial., *Invest. Ophthalmol. Vis. Sci.*, vol. 64, no. 8, p. 3754, 2023.

[187] EyePoint P. EyePoint Pharmaceuticals reports positive masked safety update for lead product candidate EYP-1901 in ongoing PAVIA and DAVIO 2 phase 2 clinical trials as of September 1, 2023. BioSpace. September 11, 2023. Accessed Septe, 2023.

[188] Adverum. Adverum provides update on ADVM-022 and the INFINITY Trial in patients with diabetic macular edema. Adverum. July 22. 2021. Accessed Dec 17, 2023., 2021. https://investors.adverum.com/news/news-details/2021/Adverum-Provides-Update-on-ADVM-022-and-the-INFINITY-Trial-in-Patients-with-Diabetic-Macular-Edema/default.aspx

[189] Kunzmann K. Gene Therapy ADVM-022 provides mixed efficacy, safety in treating DME. HCP Live. November 13, 2021. Accessed Dec 17, 2023., 2021.

[190] 4D Molecular Therapeutics. 4DMT announces first patient enrolled in 4D-150 phase 2 SPECTRA clinical trial in DME, and expansion of 4D-150 phase 2 stage in PRISM clinical trial in wet AMD | 4D Molecular Therapeutics. September 7. 2023. Accessed October 12, 2023. https://4dmt.gcs-web.com/news-releases/news-release-details/4dmt-announces-first-patient-enrolled-4d-150-phase-2-spectra/

[191] Zhou L, et al. Imatinib ameliorated retinal neovascularization by suppressing PDGFR-α and PDGFR-β. Cell Physiol Biochem. 2018;48(1):263–73. 10.1159/000491726. 30007969 10.1159/000491726

[192] Han-Soo YD. Life Science proves efficacy of diabetic treatment. KBR. May 13, 2020. Accessed September 24, 2023., 2020. https://www.koreabiomed.com/news/articleView.html? idxno = 8231.

[193] EyePoint P. EyePoint Pharmaceuticals reports positive masked safety update for lead product candidate EYP-1901 in ongoing PAVIA and DAVIO 2 phase 2 clinical trials as of September 1, 2023. BioSpace. September 11, 2023. Accessed September 24, 2023. https://www.biospace.com/article/eyepoint-pharmaceuticals-reports-positive-masked-safety-update-for-lead-product-candidate-eyp-1901-in-ongoing-pavia-and-davio-2-phase-2-clinical-trials-as-of-september-1-2023/

[194] Kita T et al. Oct., Plasma Kallikrein-Kinin System as a VEGF-Independent Mediator of Diabetic Macular Edema, *Diabetes*, vol. 64, no. 10, pp. 3588–3599, 2015, 10.2337/db15-0317 10.2337/db15-0317 PMC4587649 25979073

[195] Dugel PU, et al. Phase 1 dose-escalation study of plasma kallikrein inhibitor THR-149 for the Treatment of Diabetic Macular Edema. Transl Vis Sci Technol. Dec. 2021;10(14):28. 10.1167/tvst.10.14.28. 10.1167/tvst.10.14.28 PMC8711005 34940810

[196] Hutton D. Oxurion reaches enrollment target in clinical trial for THR-149 in DME. Ophthalmology Times. May 26, 2023. Accessed November 7, 2023., 2023.

[197] Bhatwadekar AD, Kansara VS, Ciulla TA. Investigational plasma kallikrein inhibitors for the treatment of diabetic macular edema: an expert assessment, *Expert Opin. Investig. Drugs*, vol. 29, no. 3, pp. 237–244, Mar. 2020, 10.1080/13543784.2020.1723078 10.1080/13543784.2020.1723078 PMC7265898 31985300

[198] Rezolute announces initiation of a phase 2 study of RZ402 in patients with diabetic macular edema. Rezolute, Inc. December 15, 2022. Accessed September 29. 2023., 2022. https://ir.rezolutebio.com/news-events/press-releases/detail/310/rezolute-announces-initiation-of-a-phase-2-study-of-rz402

[199] Hassan JW, Bhatwadekar AD. Senolytics in the treatment of diabetic retinopathy. Front Pharmacol. Aug. 2022;13. 10.3389/fphar.2022.896907. 10.3389/fphar.2022.896907 PMC9462063 36091769

[200] Crespo-Garcia S, et al. Therapeutic targeting of cellular senescence in diabetic macular edema: preclinical and phase 1 trial results. Nat Med. Feb. 2024;30(2):443–54. 10.1038/s41591-024-02802-4. 10.1038/s41591-024-02802-4 38321220

[201] Bhisitkul R et al. Jun., UBX1325, A Novel Senolytic Treatment for Patients with Advanced DME or wet AMD: 24-Week Results of a Phase 1 Study, *Invest. Ophthalmol. Vis. Sci.*, vol. 63, no. 7, p. 4287, 2022.

[202] Nity Biotechnology. UNITY Biotechnology announces positive 48-week results from phase 2 BEHOLD study of UBX1325 in patients with diabetic macular edema. Unity Biotechnology. April 24. 2023. Accessed October 1, 2023., 2023.

